# When international academic conferences go virtual

**DOI:** 10.1007/s11192-020-03754-5

**Published:** 2020-11-19

**Authors:** Martin Thomas Falk, Eva Hagsten

**Affiliations:** 1 Department of Business and IT, School of Business, University of South-Eastern Norway (USN), Gullbringvegen 36, 3800 Bø, Norway; 2 School of Social Sciences, University of Iceland, Reykjavík, Iceland

**Keywords:** Online conference, Virtual conferences, Video conferences, Academic conferences, High-speed broadband, Probit estimations, I20, I23, O33

## Abstract

This study investigates the extent to which international academic conferences changes format to virtual when faced by sudden Covid-19 related immobility. Data on 587 conferences in the fields of business, economics, information technology, management and other social sciences that were planned to be held between March and August 2020 are retrieved from authorised conference listings. Approximately 28% of the conferences changed to virtual format during the period of time studied. Probit estimations reveal that the probability of changing format to virtual increases with the country of location (United States), planning horizon and the available quality of broadband infrastructure in the scheduled conference country. However, the role of planning horizon differs across fields and location of the conference. The probability of virtual conferences is highest in the United States and for academic conferences in the field of information technology.

**Electronic supplementary material:**

The online version of this article (10.1007/s11192-020-03754-5) contains supplementary material, which is available to authorized users.

## Introduction

Unexpected extraordinary events such as financial crises, ash clouds or more recently, the global spread of the Covid-19 virus dramatically change the opportunities for academics from around the world to gather and meet in-person at conferences. Conferences are not only important for networking in general (Oester et al. 2017; Hansen and Budtz Pedersen 2018), but also for keeping current with trends in the field (Harrison 2010), developing potential research collaborations (Wang et al. 2017; Chai and Freeman 2019), disseminating research (Black et al. 2020), identifying job opportunities, career development (Oester et al. 2017; Black et al. 2020; Kim et al. 2020), renewing friendships (Achakulvisut et al. 2020), knowledge transfer (Goel and Grimpe 2013; Goel et al. 2014) and bringing together practitioners and academics (Dorsch et al. 2014). According to Hansen and Budtz Pedersen (2018), the preparation and the presentation of a paper and the subsequent feedback from the audience increase the quality of the research and thus the chances of publication in a high-ranking journal.

Measures taken to combat the pandemic such as travel restrictions, closed borders and gathering bans led to cancellations of many conferences, meetings and workshops and forced the scientific community to consider alternative (virtual) formats based on video conferencing software (Gichora et al. 2010; Abbott 2020; Achakulvisut et al. 2020; Arnal et al. 2020; Viglione 2020). The sharp rise in the stock exchange valuation of the main software provider for video conferencing during the pandemic reflects the strong surge in demand for its product, possibly implying that its diffusion is reaching a more general phase (Rogers 2003).^1^ In April 2020, there are Zoom Video sessions with a total of 300 million (non-unique) participants per day, compared to only 10 million in December 2019.^2^ Still, little is known about whether organisers of international academic conferences follow this trend and choose to go virtual in times of crisis.

This study investigates the extent to which international academic conferences changes format to virtual when faced by sudden Covid-19 related immobility. Special focus is put on the host city and country, the planning horizon as well as the broadband internet speed available in the planned conference country. Control variables include kind of venue, size of the conference, academic field and whether the organiser is an association. Data encompass information on 587 academic conferences in the field of social sciences meant to be held worldwide during March to end of August 2020. Probit models are used to estimate factors of importance for a change of format to virtual.

An online conference can be described as any conference where the primary medium of presentation and interaction is the World Wide Web (WWW) (Thatcher 2006; Gichora et al. 2010). This includes video conferences, teleconferences, chat rooms and intranet discussions. Web-based video conferencing is increasingly used in education and in business from the mid-1990 s onwards (Daly-Jones et al. 1998; Lawson et al. 2010). Virtual conferences are also commonly seen as a solution to reduce the carbon footprints of travel to academic and business conferences (Abbott 2020). Many powerful web conferencing software and systems are now available (Adobe connect Webinars, WebEx Premium, GoToWebinar Seminar, Zoom, Google Hangouts, Skype, Apache and Team Viewer) (see Peuler and McCallister 2019 for an overview). In this analysis, a conference is considered virtual when it puts some of its content in an online window or is held entirely in real time by use of video conferencing software.

This study contributes a first empirical investigation of how organisers of international academic conferences proceed when they are faced with whole groups of participants that are no longer mobile and thus cannot actively network in-person. The unique dataset at hand encompasses a large representative sample of conferences in the fields of social science and information technology. Results of the Probit estimations indicate a certain resistance towards virtual conferences, although with more time for planning and with other facilitating factors such as broadband access of decent quality a gradual adjustment to the actual situation appears. This adjustment is fastest in the United States and follows the typical path of diffusion of an innovation (Rogers 2003).

The study is structured as follows: section “Conceptual background” introduces the conceptual background and provides the hypotheses, section “Empirical model” presents the empirical model while section “Data” describes the dataset. The empirical results are revealed in sections “Empirical results” and “Conclusion” concludes.

## Conceptual background

Holding virtual meetings is not a new phenomenon and in recent years this has been facilitated by improved communication technologies and higher broadband speed. The benefit of virtual conferences is highlighted in relation to environmental concerns about the carbon footprint created by conference travellers (Høyer and Næss 2001; Nevins 2014; Abbott 2020; Blackman et al. 2020). Another advantage of virtual conferences is that there is no real limitation of the total number of participants (Parncutt and Seither-Preisler 2019). A virtual format also allows pre-recorded presentations (Chen et al. 2020; Zhang et al. 2020), something that might be especially useful when the conference includes participants from different time zones. Virtual conferences may also open the door to international research discussions for participants from poorly funded countries (Fraser et al. 2017).

Just like physical meetings, organising an online conference is not an easy task (Gichora et al. 2010; Oester et al. 2017), although it may raise other issues such as coordination across time zones (Olson and Olson 2000; Oester et al. 2017). Broadband access and usage are other critical factors that must be managed to safeguard against conference interruptions and provide practical platforms for the participants (Gichora et al. 2010). Earlier studies report that video conferencing tools are extremely inconvenient (Olson and Olson 2000) and Fraser et al. (2017) reckon that the technology is still unreliable.

Often, a large part of the networking at conferences takes place during coffee breaks, in corridors or at the organised dinner. Virtual conferences cannot offer this kind of communication from which sometimes entire research projects have emerged (Harrison 2010; Oester et al. 2017). Fraser et al. (2017) concludes that a main limitation of the purely virtual conferencing model is that it cannot replicate face-to-face networking. Up to now, distance meetings are usually designed to efficiently achieve a goal or to solve specific problems, following a strict agenda. Another aspect is the capacity to absorb, Rowe and Frewer (2000) as well as Abelson et al. (2003) show that learning and collaboration are better achieved through mutual two-way exchange, which is more problematic in the virtual format. Interactive virtual conferencing may accommodate a limited number of people, hundreds of participants cannot communicate with each other. Virtual meetings also cannot guarantee that people will participate actively (Oester et al. 2017).

Issues surrounding technology as well as the missed opportunity for informal interactions could well contribute to the reason why the use of online conferencing is low. Another possible aspect is related to “academic tourism” (Thatcher 2006). Conferences are often held in cities with cultural heritages and tourist attractions (such as London and Paris). In the sample studied, planned conference cities include not only London and Paris, but also Berlin, Barcelona, Chicago, Rome and Venice. A virtual conference cannot be a substitute for the live experience of being in one of the major cultural cities.

The decision of conference organisers to change format to virtual instead of cancelling or postponing may be embedded within the Unified Theory of Acceptance and Use of Information Technology (UTAUT) developed by Venkatesh (2000) and Venkatesh et al. (2003). According to this theory, information system use depends on a bundle of factors including performance expectancy, effort expectancy, social influence and facilitating conditions. The first three factors relate to intended behaviour while the fourth determines the usage. The intentions are difficult to measure or can be estimated only indirectly, although facilitating aspects such as conference and location specifics, including access to high-speed broadband internet are available.

Many, including conference organisers, were taken by surprise when the Covid-19 virus rapidly began its tour around the world. On short notice, it is difficult to change the format of a gathering. Conference organisers possibly also lack information about the acceptance of a virtual substitute by presumptive participants. However, given time, more investigations may be made and planning of an alternative format can be carried out. This means that the planning horizon is a crucial factor that influences the decision to change format to virtual. In this study, the sample period spans from March to end of August 2020. Under normal circumstances, this would be considered an extremely short planning time for a substantial change of conference format. Gichora et al. (2010) suggest that between six and nine months are needed for a proper re-organisation of the conference, including the setup of technology. Given the abrupt unfolding of events and the short time frame at hand for changes, the first hypothesis states that the likelihood of the conference organiser to change format to virtual is low:

### **H1**

The proportion of virtual conferences is low.

Over time, however, with better opportunities for planning, this might become more likely, leading to the second hypothesis:

### **H2**

The proportion of virtual conferences increases with planning time.

Besides planning horizon, several other factors might play a role in the decision to change format to virtual. One of them is the country where the conference is planned to take place. Large geographical distances within a country, such as the United States, may inspire to frequent use of virtual conferencing. With many patents in the field of technologies relating to video conferencing (e.g. Nayak 2020) and with several major software companies based there (Peuler and McCallister 2019), the United States could possibly even be a typical lead market (as defined by Beise 2004) for video conferencing software. This leads to the third hypothesis:

### **H3**

The probability of changing format to virtual conference is host country specific.

A necessary condition for virtual meetings is the availability of decent underlying infrastructure such as high-speed broadband internet in the host country. There are large variations across the world in available broadband speed with a factor 25 difference between the fastest and the slowest country.^3^ This constitutes the fourth hypotheses:

### **H4**

The probability of changing format to virtual conference increases with broadband speed.

The control variables include other conference-specific factors such as the duration of the conference, its venue and focus (field or main topics). Hansen et al. (2020) identify four different dimensions of academic events: size, focus, participants and tradition. Size can be measured by the number of days of the conference. Those that span several days are likely to be larger and more difficult to convert to a virtual format than smaller one-day conferences. Another important feature is whether the conference is held annually by an association or if it is independent. The former organiser may have a longer tradition than the latter. The kind of venue could also be a factor in the decision. Typical venues are universities, international organisations, hotels or conference centres. Organisers who have a contract with an external provider of facilities might prefer to postpone the conference until next year. Otherwise, cancellation fees may be incurred that are difficult to cover. Capital city is an additional control variable. Such cities are centres of science and home to headquarters of multinational companies and their inhabitants are usually advanced users of technology. Because of this it can be expected that conferences planned to be held in a capital city more easily can change format to virtual.

## Empirical model

Given the theoretical considerations highlighted in the conceptual background, the decision to change format to virtual instead of cancelling or postponing the conference is not random and depends on a bundle of characteristics relating to the organisers and the location. Probit or Logit models can be used to estimate the choice of individual organisers, based on the random utility model (Ben-Akiva and Lerman 1985). This model assumes that each category represents a different utility level. The utility of each alternative depends on observable und unobservable attributes. Observed attributes are represented in the utility function by explanatory variables.

A series of assumptions about four elements constitutes the discrete choice framework (Ben-Akiva and Bierlaire 1999): (1) the decision maker is the person or agent making a choice, (2) the decision maker must choose from the set of available alternatives that are mutually exclusive and collectively exhaustive (3), the alternatives have characteristics that make them attractive to the decision maker and (4) the decision maker evaluates the attractiveness of all alternatives. In the model of random utility maximization, the decision maker selects the alternative that maximizes his or her (expected) utility (net profit, profits, satisfaction). Under the random utility maximization hypothesis, it can be assumed that the conference organiser chooses the alternative which offers maximal utility.

In this analysis, the conference organiser faces one of two possible outcomes: i) cancellation or postponement or ii) virtual conference. These two alternatives provide the organiser with a given utility. For each conference *i*, the status *Y* equals to 1 can be observed if the conference changes format to virtual and 0 if it does not. The outcome is assumed to be the unobserved latent variable Y^*^, observed as the binary variable *Y*:

1 $$Y = \left\{ {\begin{array}{*{20}l} 1 \hfill & {\text{if}} \hfill & {Y^{*} > 0} \hfill \\ 0 \hfill & {\text{if}} \hfill & {Y^{*} \le 0} \hfill \\ \end{array} } \right..$$

The Probit model links the observed decision to the unobserved probability of changing format to virtual with the underlying characteristics *X* via a standard normal cumulative distribution function ($\varphi$) (with the individual index *i* suppressed for convenience) as follows (Wooldridge 2013):

2 $$\Pr \left( {Y = 1|X} \right) = \varphi \left( {X^{{\prime }} \beta } \right),$$

And where the likelihood to change format to virtual is a function of the observable characteristics *X*:

3 $$Y^{*} = X^{{\prime }} \beta + \varepsilon .$$

Vector *X* consists of covariates with characteristics of the planned conference and its scheduled location and *ß* is the corresponding vector of coefficients. Random and unobservable factors influencing the decision are captured by the error term $\varepsilon$. The relationship can be estimated by the Probit model using the maximum likelihood estimator. Standard errors are clustered at the city level because the error term of conferences held in the same city may not be independent of each other.

Based on the theoretical considerations and the literature outlined above, the probability that a conference changes format to virtual $Y^{*}$ is specified as a function of the ensuing factors:

4 $$\begin{aligned} Y_{i}^{*} & = \alpha_{0} + \alpha_{1} { \ln }(BROADBAND)_{i} + \alpha_{2} TIME_{{}} + \alpha_{3} TIME^{2} + \alpha_{4} { \ln }(BROADBAND)_{i} x TIME \\ & \quad + \alpha_{5} ASSOCIATION_{i} + \alpha_{6} VENUE_{i} + \mathop \sum \limits_{F = 1}^{6} \beta_{F} FIELD_{iF} + \mathop \sum \limits_{T = 1}^{4} \beta_{T} TYPE_{iT} + \mathop \sum \limits_{S = 1}^{3} \beta_{S} SIZE_{iS} \\ & \quad + \mathop \sum \limits_{C = 1}^{3} \beta_{2C} COUNTRYGROUP_{iC} + \alpha_{7} CAPITALCITY_{i} + \varepsilon_{i} , \\ \end{aligned}$$

where *i* denotes the planned conference for a given starting date and *ln()* is the natural logarithm. The constant is represented by $\alpha_{0}$ and $\varepsilon_{i}$ is the error term. Variable *BROABAND* measures the broadband speed, *TIME* indicates number of days from the beginning of March to the planned start of the conference, *ASSOCIATION* is a dummy variable equal to one if the conference is annually held by an association and zero otherwise. *VENUE* is a dummy variable equal to one if the conference site is a hotel or convention centre and zero if it is not. Dummy variables for *FIELD* and kind of conference *TYPE* are also included. The length of the conference is measured by *SIZE*, *COUNTRYGROUP* is a set of country group dummy variables and *CAPITALCITY* is a dummy variable equal to one if the conference is planned to be held in the capital. The specification also includes an interaction term between planning horizon and broadband speed, *BROADBAND*×*TIME*. In order to allow for a non-linear form, the quadratic planning horizon $TIME^{2}$ is encompassed.

An alternative to the standard Probit model with cluster-adjusted standard errors at the city level is to estimate a two-level random-intercept model (Skrondal and Rabe-Hesketh 2004) where the error term may vary across cities and, thus, accounts for unobserved time-invariant city factors such as attractiveness.

## Data

Data for the analysis consists of information on 587 academic conferences around the world in the fields of economics and management, banking and finance, business, other social sciences (political science, sociology and human geography) as well as data sciences and information technology from various sources. One extensive source is the listing of conferences, workshops, meetings and training schools by “Resources for Economists” (American Economic Association, AEA) and EconBiz (https://www.aeaweb.org/resources/conferences-meetings). This database encompasses information on almost 25,000 economic and business events around the world. Future events are published throughout the year, and there is also information about past meetings. The AEA database is mainly oriented towards business, economics and policy, conferences in the fields of management, data sciences, information technology as well as other social sciences are added to the database. These latter additions are listed in two popular conference marketing tools “Easychair” and “Conference Maker”. In addition, conferences in the field of social science listed in the conference calendar by Routledge are used (https://www.routledge.com/conferences) together with the AAG (American Association of Geographers) conference calendar (http://www.aag.org/cs/calendar_of_events).

A particular problem with the listing of events is the existence of predatory or sham conferences (Lang et al. 2019; Sonne et al. 2020). These kinds of conferences are not included in the analysis. Criteria used to exclude less serious conferences are the reputation of the scientific advisory board (established researchers who have published in SSCI-journals and presence of an international scientific committee), the existence of an underlying association journal and presence of a university or research institution web site for additional information. Being listed by the American Economic Association is normally also in itself a sign of quality. It is important to note that the selected number of conferences, although large, are only a subset of the meetings that were meant to take place during this period. Many conferences in the social sciences are announced by invitation only and not by a “Call for a Paper”.

Academic conferences and meetings included in the analysis are selected for the period 2 March to 31 August 2020. The start date relates to the point in time when the first conference was changed. Although a certain awareness of the crisis, before mid-March, several events still took place, but from then onwards, all conferences have been either cancelled, postponed or changed format to virtual due to government laws, travel restrictions, bans of large gatherings and border closures. In the majority of postponements, the event is planned for the same period next year while virtual conferences are normally held at the scheduled date, implying that these two alternative actions are mutually exclusive. Those conferences that were actually held as planned in March are excluded from the dataset.

Information about the planned conference, such as venue (hotel, congress centre or university) is retrieved manually from its website (conferences included in the analysis and their format are listed in Online Data Appendix). Generally, this website also indicates whether it is an annually held conference by an association or if it is independent. Kind of conference is identified by use of keywords on the website, the call for papers or information about the underlying association. Other information available is country of the conference, city and kind of conference. Information on broadband speed is provided by Howdle (2020) and refers to the period May 2019 to April 2020.

Somewhat more than one out of four conferences changed format fully or partially to virtual, implying that the first hypothesis cannot be rejected. From June 2020, some conferences are held as hybrids, which means that they offer a combination of virtual and personal attendance. These conferences are defined as virtual, as are those with pre-recorded presentations. The moderate share of virtual conference is consistent with the pre-Covid-19 studies by Fraser et al. (2017), Hamm, Frew and Lade (2018) and Sarabipour et al. (2020).

Conferences in the fields of economics and economic policy account for 57% of the total amount (Table 1). The United States, the EU/EEA countries and other non-European OECD countries account for 89% of the conferences.^4^ This unequal distribution of international academic conferences is consistent with the evidence for the number of scientific publications, which also shows a clear underrepresentation in the social sciences of developing and emerging countries (Alatas 2003). Highly popular conference cities are Barcelona (16 conferences), Berlin, London and Paris (13 each), Chicago (11) and Brussels (10). Annually planned association conferences are most common (87.5%) and university or research institutions are preferred venues. A typical duration is two days (35.3%) and traditional conferences account for 60.6%, followed by workshops with 16.0%. Average broadband download speed is 51 Mbps. The planning horizon, measured as days from March (2^nd^) when the first conference was altered, is 95 days on average.

**Table 1:** Descriptive statistics

	Mean/percent		Mean/percent
Virtual conferences	28.2	Association conference	87.5
*Field*		Venue Hotel/conference centre	21.8
Banking & Finance	7.5	*Length of conference*	
Business	8.5	Number of days = 1	6.7
Data science and Information technology	14.7	Number of days = 2	35.3
Economics & Policy	56.8	Number of days = 3	31.4
Management	3.9	Number of days = 4+	26.6
Human geography, sociology, political science	8.5	*Type of conference*	
Mean download speed 2019/2020(Mbps)	51.3	Conference	60.6
Time (Days from March)	95.1	Workshop	16.0
*Country group*		Training school	7.7
US	15.2	Other	15.7
EU-EEA (European Economic Area)	63.8		
OECD non-Europe	9.9		
Other countries	11.1		
Capital city	30.2		

*Source*: See text

The proportion of virtual conferences is highest in the United States (46%), with time for planning and within certain fields such as information technology. Association conferences, larger conferences, conferences planned for capital cities and in countries with above average broadband speeds also change format more often to virtual (Table 2).

**Table 2:** Proportion of virtual international academic conferences by characteristics (per cent)

*Broadband download speed 2019/2020*		*Venue University or research organisation*	27.2
< 37.8Mbps	23.9	Venue hotel or convention centre	32.0
≥ 37.8Mbps X < 51.3Mbps	25.2	*Field*	
≥ 51.3Mbps < 71.3Mbps	22.7	Banking Finance	9.1
≥ 71.3Mbps	40.2	Business	24.0
*Planning horizon*		Information technology	50.6
X < 70.5 days	15.0	Economics and policy	25.4
< 70.5 days X < 98 days	15.6	Management	26.1
< 98 days X < 119	27.7	Social science	32.0
X > 119 days	52.8	*Number of planned days*	
*Country group*		1	28.2
US	46.1	2	21.6
EU+EEA	25.5	3	28.3
OECD non-Europe	25.9	4+	37.6
Other	23.1	*Type*	
Other cities	26.8	Conference	29.6
Capital city	32.0	Workshop	23.4
Non-association conference	23.3	Training school	24.4
Association conference	29.1	Other	30.9

*Source*: See text

## Empirical results

Probit estimations reveal that the probability of a scheduled international academic conference changing format to virtual instead of being cancelled or postponed depends on the country of location (United States), the planning horizon, the availability of high-speed broadband and the field (Table 3). Three specifications are provided: (i) a basic Probit model including all variables (ii), a specification augmented by the quadratic term to allow a non-linear format for the planning time and (iii) the final specification excluding the insignificant variables. The Probit estimations are based on cluster adjusted standard errors at the city level. According to the Likelihood ratio test (LR), the Multi-level model with random city effects is rejected at the 5% level. Therefore, the interpretation of the results focuses on the standard Probit estimations exclusive of the insignificant variables (Specification iii) and the results of the Mixed-Effects Probit model are not reported.

**Table 3:** Probability of international academic conferences changing format to virtual

	(ii)		(ii)		(iii)	
	Coeff.	*z*-stat	Coeff.	*z*-stat	Coeff.	*z*-stat
Banking Finance (ref Economics & Policy)	− 0.439	− 1.40	− 0.409	− 1.34	− 0.370	− 1.22
Business	0.079	0.34	0.125	0.56	0.178	0.81
Management	0.339	1.14	0.377	1.29	0.442	1.56
Social science	0.449*	1.94	0.438*	1.88	0.482**	2.09
Data science, Information technology	0.528***	3.07	0.506***	2.85	0.559***	3.14
Time (Days from 2 March)	0.013***	7.64	− 0.0010	− 0.18	− 0.001	− 0.09
Time squared (Days from 2 March)			0.0001***	2.73	0.000***	2.67
Log broadband speed in 2019/2020	0.159**	2.02	0.156**	1.97	0.146*	1.84
United States (ref. Non-US)	0.894***	4.78	0.834***	4.50	0.841***	4.69
Capital city	0.254*	1.91	0.249*	1.84	0.252*	1.85
Association conference	0.082	0.40	0.076	0.39		
Venue Hotel/conference centre	− 0.144	− 0.86	− 0.168	− 1.01		
Size: Number of days = 2 (ref=1)	− 0.216	− 0.85	− 0.182	− 0.72		
Number of days = 3	− 0.093	− 0.33	− 0.049	− 0.17		
Number of days = 4+	− 0.013	− 0.05	0.028	0.10		
Conference	0.045	0.28	0.092	0.56		
Workshop	− 0.057	− 0.23	− 0.014	− 0.06		
Training school	− 0.239	− 0.69	− 0.215	− 0.62		
Constant	− 2.814***	− 5.96	− 2.260***	− 4.38	− 2.273***	− 5.24
Number of observations	587		587		587	
Number of groups (cities)	282		282		282	
McFadden Pseudo *R*^2^	0.205		0.196		0.206	
Log pseudolikelihood	− 276.8		− 280.0		− 278.8	
LR test ME Probit versus probit model (*p* value)	0.245		0.246		0.226	
Wald test time, time squared=0 (*p* value)	0.00				0.00	

	Marginal effects					
	d*y*/d*x*	*z* stat	d*y*/d*x*	*z* stat	d*y*/d*x*	*z* stat
Banking finance (ref Economics & Policy)	− 0.118	− 1.40	− 0.108	− 1.33	− 0.098	− 1.21
Business	0.021	0.34	0.033	0.56	0.047	0.81
Management	0.091	1.15	0.100	1.31	0.117	1.58
Social science	0.121**	1.98	0.116*	1.91	0.128**	2.14
Data science, Information technology	0.142***	3.19	0.134***	2.95	0.149***	3.30
Time (Days from 2 March)	0.004***	9.48	0.004***	9.72	0.004***	10.04
Log broadband speed in 2019/2020	0.043**	2.03	0.041**	1.97	0.039*	1.84
United States (ref. non-US)	0.241***	4.98	0.220***	4.69	0.224***	4.93
Capital city	0.068*	1.94	0.066*	1.87	0.067*	1.89
Association conference	0.022	0.40	0.020	0.39		
Venue hotel/conference centre	− 0.039	− 0.86	− 0.044	− 1.00		
Size: Number of days = 2 (ref.=1)	− 0.058	− 0.85	− 0.048	− 0.72		
Number of days = 3	− 0.025	− 0.33	− 0.013	− 0.17		
Number of days = 4+	− 0.003	− 0.05	0.007	0.10		
Conference	0.012	0.28	0.024	0.56		
Workshop	− 0.015	− 0.23	− 0.004	− 0.06		
Training school	− 0.064	− 0.70	− 0.057	− 0.62		

Asterisks ***, **, * denote significance at the ¨1, 5 and 10% levels, respectively. This table reports the marginal effects, d*F*/d*x*, and the corresponding *z* values. *Z*-stat in the Probit model is based on cluster-adjusted standard errors at the city level (282 clusters). The standard Probit and the mixed-effects (ME) Probit models are both estimated using Stata 15.1, procedures Probit and MEProbit, the latter with random city effects

The dummy for capital city is positive and significant at the 10% level indicating that conferences planned for these locations are more likely to change format to virtual. Size (number of days) of the planned conference, kind of venue, type of conference (conference, workshop, training school) and whether the conference is related to an association are all not significant. The pseudo R-squared (goodness of fit) is 0.20. In addition, 85% of the conference observations are correctly classified (with a cut off value of 0.5), adding to the conclusion in the data section that Hypothesis 1 cannot be rejected.

**Fig. 1:**
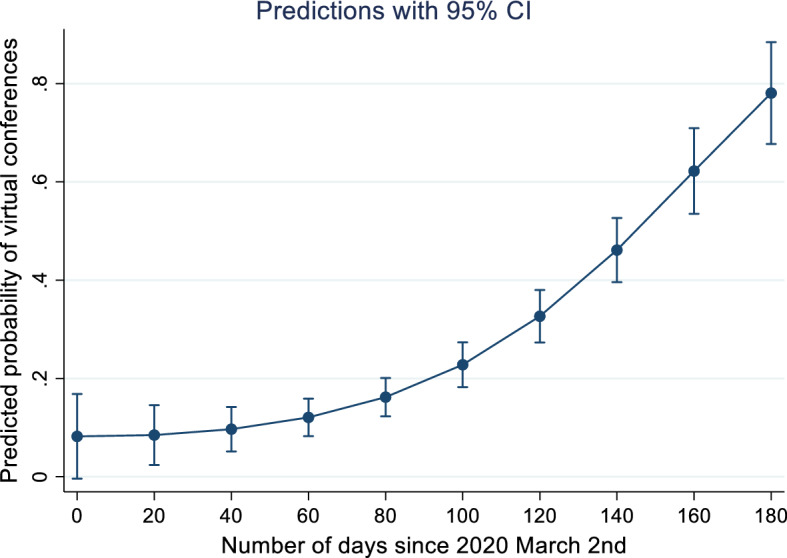
Relationship between planning horizon and the probability of changing format to virtual. *Notes*: Predicted probabilities are calculated based on the Probit estimates displayed in Table 3. CI means confidence interval

Conferences in the United States have a 22 percentage points higher probability of changing format to virtual compared to the reference group (Non-United States) (Table 3 lower panel). This is substantial given that the average percentage of virtual conferences is 28.2%. In contrast, the probability of changing to virtual in the EU/EEA or OECD non-Europe countries is not significantly different from the reference group and thus not included in the final specification. The impact of planning horizon is also relatively large. There is a non-linear relationship between the planning horizon and the likelihood to change format to virtual. The squared term is positive and highly significant indicating a convex relationship (Specification ii). After 100 days the probability increases by 15 percentage points and towards the end of the sample period the surge is 70 percentage points (Fig. 1), that is, a pattern resembling the typical diffusion of innovation curve (Rogers 2003). Therefore, neither the second nor the third hypothesis can be rejected. In addition, broadband speed has a marked effect, satisfying the fourth hypothesis. An increase in one standard deviation of the logarithm of broadband speed (from 51 mbps which is the level of France or Canada to 76 mbps reflecting the level of United States or Singapore) leads to an increase in the probability to go virtual by 2.7 percentage points ceteris paribus (marginal effect of 0.039 multiplied by one standard deviation of log download speed which is 0.698). Academic field of the conference is also relevant. Social sciences as well as data science and information technology have between 13 and 15 percentage points higher probability of changing format to virtual.

**Fig. 2:**
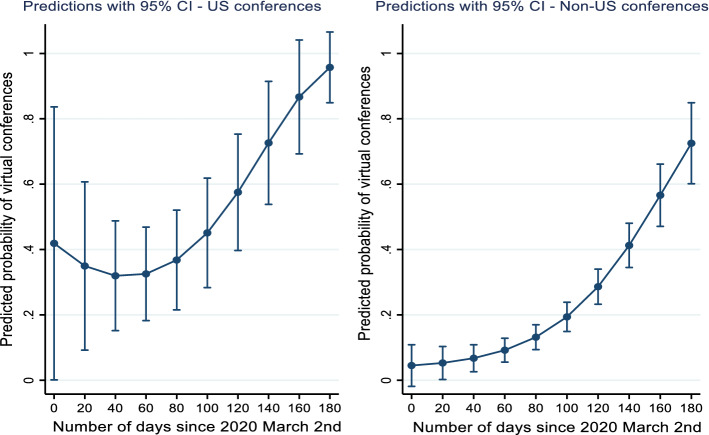
Relationship between planning horizon and the probability of changing format to virtual by scheduled location. Notes: Predicted probabilities are calculated based on separate Probit estimates for US and Non-US conferences

**Fig. 3:**
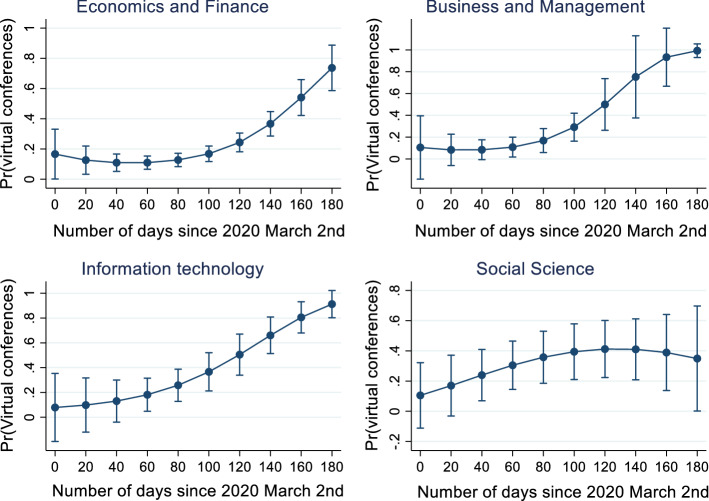
Relationship between planning horizon and the probability of changing format to virtual by academic field. Source: Based on separate Probit estimates by field based on specification iii and excluding field dummy variables in Table 3

The estimations also show that the probability of changing format to virtual is much higher for conferences scheduled in the United States than in other countries, and this can be observed over the entire period. Three months after the start of the crisis, the probabilities begin to increase at an exponential rate in all locations (Figs. 2, Appendix). Estimations by subfields reveal that the relationship between planning horizon and the decision to change to virtual format is more relevant for information technology, economics and policy as well as management and business conferences. At the end of the sample period almost all conferences in information technology and business and management change format to virtual (equal to a predicted probability of 100%) (Fig. 3, Appendix). In contrast, in social sciences the likelihood stagnates at the level of 40%.

**Fig. 4:**
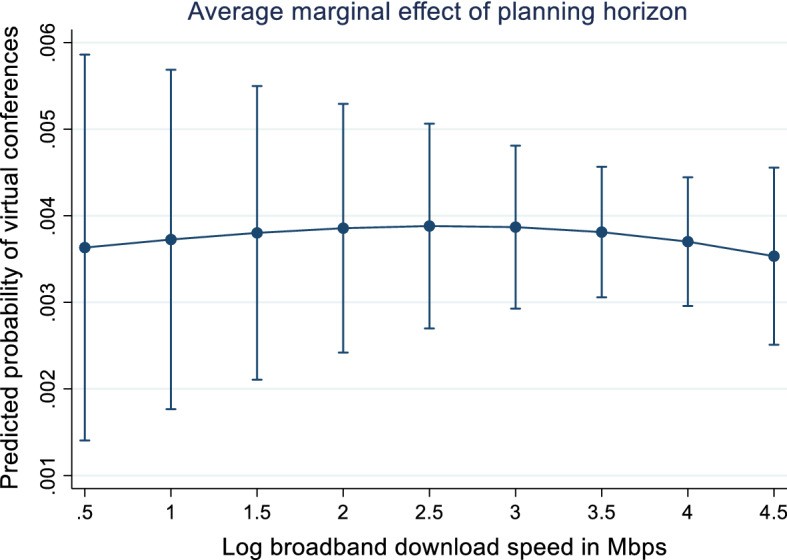
Combined effect of broadband speed and planning horizon on the probability of changing format to virtual. Source: Based on the estimates in Table 4

Several robustness checks are provided. There is, for instance, a possibility that access to decent broadband capacity is an underlying facilitator. Because of this, two interaction terms are added to the specifications: (i) between broadband speed and planning horizon and (ii) between broadband speed and the planning horizon squared. Estimations including these interaction terms reveal that the significance of the planning horizon increases for conferences scheduled for locations with high broadband speed (Table 4 and Fig. 4, Appendix). The five broadband and planning time variables are jointly significant at the one per cent level using the Likelihood-ratio (LR) test, although the interaction terms do not give substantially new insights to the analysis. Second, estimations are conducted for the early phase (March to June). However, the significance of the main variables (field, broadband speed and the US country dummy variable) do not change much (see Table 5, Appendix, for the results from March to June).

**Table 4:** Probability of international academic conferences changing format to virtual, including interaction term

	Coeff	*z*-stat
Banking Finance (ref Economics & Policy)	0.384	− 1.26
Business	0.174	0.78
Management	0.426	1.50
Social science	0.485**	2.10
Data science, Information technology	0.555***	3.12
Time (Days from 2 March)	0.019	− 0.71
Time squared (Days from 2 March)	0.000	1.45
Log broadband speed	0.035	0.10
Time (Days from 2 March X Log broadband speed	0.005	0.69
Time squared (Days from 2 March) X Log broadband speed	0.000	− 0.91
United States (ref. Other)	0.830***	4.69
Capital city	0.265*	1.94
Constant	1.845	− 1.33
Number of observations	587	
Number of groups (cities)	282	
McFadden Pseudo *R*^2^	0.204	
Log pseudolikelihood	− 278.3	
Wald test time, time squared, Log broadband speed and interaction terms = 0 (*p* value)	113.9	

Asterisks ****, **, * denote significance at the 1%, 5% and 10% levels, respectively. This table reports the coefficients and the corresponding *z* values obtained from a Probit model. *Z*-stat in the Probit model is based on cluster-adjusted standard errors at the city level (282 clusters)

**Table 5:** Probability of international academic conferences changing format to virtual (March to June 2020)

	Coef.	*z*-stat	d*y*/d*x*	*z* stat
Banking Finance (ref Economics & Policy)	− 0.266	− 0.85	− 0.064	− 0.85
Business	0.196	0.83	0.047	0.83
Management	0.505*	1.73	0.121*	1.76
Social science	0.702***	3.05	0.168***	3.20
Data science, Information technology	0.619***	2.76	0.149***	2.88
Time (Days from 2 March)	− 0.002	− 0.13	0.003***	2.89
Time squared (Days from 2 March)	0.000	0.87		
Log broadband speed	0.230**	2.23	0.055**	2.25
United States (ref. Other)	0.869***	4.15	0.209***	4.34
Capital city	0.363**	2.27	0.087**	2.33
Constant	− 2.657***	− 4.91		
Number of observations	450			
Number of groups (cities)	248			
McFadden Pseudo *R*^2^	0.124			
Log pseudolikelihood	− 193.54			
Wald test time, time squared=0 (*p* value)	0.00′			

Asterisks ***, **, * denote significance at the ¨1, 5 and 10% levels, respectively. This table reports the marginal effects, d*F*/d*x*, and the corresponding *z* values. *Z* stat in the Probit model is based on cluster-adjusted standard errors at the city level (248 clusters)

## Conclusions

This study investigates the extent to which international academic conferences changes format to virtual when faced by sudden Covid-19 related immobility. The empirical analysis is based on a novel database of 587 conferences planned to be held during the period of March to August 2020 within the fields of social sciences, economics, business, management and information technology. On average, 28% of the conferences change format to virtual, fully or partially, and this proportion is increasing to 78% towards the end of the sample period, measured as predicted probabilities. Probit estimates show that the likelihood of changing format to virtual mainly depends on the country of location, planning horizon, broadband speed and academic field.

In the United States, the probability of changing to a virtual conference format is 22 percentage points higher than in other parts of the world. There is also evidence that the probability of changing format to virtual will increase exponentially with more time for planning. In addition, conferences planned for countries with access to high-speed broadband are more likely to change format. The likelihood of changing format to virtual vary considerably across academic fields and is highest in data sciences, information technology, and social sciences, while economics (including policy) as well as banking and finance are lagging behind. Characteristics such as conference venue (hotel or university), size or kind of organiser are all variables not significant.

Typically, the increase of virtual conferences towards the end of the time period studied indicates that the spread of the underlying innovation (video conferencing tools) is following a steep diffusion path, although it is still unclear to what extent this will continue. The reasons why not all conferences change format to virtual, especially at the beginning of the pandemic (March to May), are partly explained by the model, but very likely there are several non-measurable aspect such as a lack of interest shown by delegates as well as organisational issues at stake. Certain adjustment costs are also expected to be at play. Standard academic conferences in the social sciences consist of keynote speeches and parallel sessions on the selected conference topics. The latter generally means presentations of between 15 and 20 min. In principle, these presentations can be held virtually. However, keeping such parallel design including two-way interactions between the speakers and the audience are more demanding (and very likely costly). It is also possible that the conference organisers believe that the acceptance and willingness to pay might be low because of the lack of social interaction and opportunities of active networking, factors normally highly appreciated by participants and supported by publishers and institutions.

The low proportion of virtual conferences also reflects the fact that organisations were taken by surprise by how events unfolded and were not at all prepared for an alternative format. This would mean that a larger degree of flexibility could be needed in the planning of future conferences, something that might require increased budgets. Thus, it is possible that large scale events will be replaced by smaller workshop-like or hybrid-style conferences in the short or medium term, where some participants appear in person and others not.

Some limitations of this study should be noted. The analysis is restricted to social sciences and information technology. There is a need for further research, including other areas such as natural sciences and humanities. Another weakness of the study is that the time span of six months is rather short and does not cover a yearly cycle of academic conferences. There is most likely also unmeasured variables of importance that affects how conference organisers act, such as perceptions and demand for virtual conferences. There are several directions for future research. One suggestion is to include additional fields, another is to extend the period of time to allow presumptive long-term changes.

### Electronic supplementary material

Below is the link to the electronic supplementary material.

Supplementary material 1 (DOCX 135 kb)

## Appendix

See Figs. 2, 3, 4, Tables 4, 5.

## Footnotes

1 The share prices for Zoom increased from January to July 2020 from USD 74 to USD 260 which is equal to an increase of 250% (https://finance.yahoo.com/quote/ZM/history?period1=1565913600&period2=1597536000&interval=1d&filter=history&frequency=1d. Accessed 15 Aug 2020).

2 https://www.nasdaq.com/articles/3-reasons-zoom-videos-impressive-rally-might-soon-end-2020-05-02. Accessed 15 Aug 2020.

3 The broadband speed in 2020 is highest in Luxembourg and Iceland and lowest in Egypt (Howdle 2020).

4 EU: Austria, Belgium, Bulgaria, Croatia, Cyprus, Czech Republic, Denmark, Estonia, Finland, France, Germany, Greece, Hungary, Ireland, Italy, Latvia, Lithuania, Luxembourg, Malta, the Netherlands, Poland, Portugal, Romania, Slovakia, Slovenia, Spain and Sweden, EEA: Iceland, Norway, Switzerland and the United Kingdom and other OECD countries: Australia, Canada, Chile, Israel, Japan, Mexico, South Korea, New Zealand and Turkey.
