# Supplementary material for: When international academic conferences go virtual
Source: Scientometrics. 2020 Nov 19;126(1):707–24. doi: 10.1007/s11192-020-03754-5 (PMC7676402; doi:10.1007/s11192-020-03754-5)
Supplement: Supplementary file 1 — Supplementary material 1 (DOCX 135 kb) [file 11192_2020_3754_MOESM1_ESM.docx]

Online data appendix

Table A1 Name of the conferences and virtual status

| Conference name | Planned location | start date | end date | Virtual =1 |
| --- | --- | --- | --- | --- |
| Campus for Finance - WHU Private Equity Conference 2020 - Otto Beisheim School of Management | Vallendar | 12/03 | 13/03 | 0 |
| MBA World Summit - Frankfurt School of Finance & Management | Frankfurt am Main | 18/03 | 20/03 | 0 |
| AEFP 45th Annual Conference 2020 "Toward a Meaningful Impact through Research, Policy, and Practice" - | Fort Worth | 19/03 | 21/03 | 1 |
| Workshop "The Dynamics of Inclusive Finance" - European Association for Banking and Financial History | Utrecht | 20/03 | 21/03 | 0 |
| 16th European Winter Finance Summit 2020 | Zermatt | 22/03 | 25/03 | 0 |
| 23rd Annual Meeting of the Swiss Society for Financial Market Research (SGF Conference) 2021 | Zürich | 25/03 | 26/03 | 0 |
| 2020 Napa/Sonoma Conference - Financial Management Association International FMA | Napa Valley | 27/03 | 28/03 | 0 |
| conference A Hundred Years of the Modern Foreign Exchange Market in London | London | 27/03 | 27/03 | 0 |
| 13th Swiss Winter Conference on Financial Intermediation 2020 | Lenzerheide | 29/03 | 01/04 | 0 |
| 19th Colloquium on Financial Markets: Asset Management | Cologne | 30/03 | 30/03 | 0 |
| 6th International Conference on Applied Theory, Macro and Empirical Finance - AMEF 2020 | Thessaloniki | 13/04 | 14/04 | 0 |
| Annual Conference of the Agricultural Economics Society 2020 | Leuven | 15/04 | 17/04 | 0 |
| NCCC-134 Committee on Applied Commodity Price Analysis, Forecasting, and Market Risk Management 2020 Annual Conference | St. Louis | 20/04 | 21/04 | 0 |
| Adam Smith Workshop in Asset Pricing & Corporate Finance 2020 | Nice | 23/04 | 24/04 | 0 |
| DIW-Workshop "Finance and Development" 2020 - German Institute for Economic Research | Berlin | 23/04 | 24/04 | 0 |
| 7th Annual Conference on the Banking Union | Frankfurt am Main | 06/05 | 06/05 | 0 |
| Fifth European Workshop on Household Finance 2020 | Stockholm | 07/05 | 09/05 | 1 |
| Conference on Financial Regulation 2020 | Chicago | 08/05 | 09/05 | 0 |
| 21th annual NOITS conference (Nordic International Trade Seminar) | Reykjavik | 14/05 | 15/05 | 1 |
| 2020 Workshop on Monetary and Financial History | Chicago | 14/05 | 15/05 | 0 |
| Tenth Anniversary FMA Applied Finance Conference - Financial Management Association International | New York | 15/05 | 25/05 | 0 |
| 12th Asia/Pacific Conference 2020 - Financial Management Association International | Nanjing | 20/05 | 22/05 | 0 |
| 37th Spring International Conference of the French Finance Association 2020 | Nantes | 25/05 | 27/05 | 0 |
| SAAMA 2020 Conference SAAMACONF-2020 | Stellenbosch | 25/05 | 27/05 | 0 |
| 8th International Symposium on Environment and Energy Finance Issues (ISEFI-2020) | Paris | 28/05 | 29/05 | 0 |
| 10th Conference on Professional Asset Management 2020 | Rotterdam | 28/05 | 29/05 | 0 |
| 2020 Financial Intermediation Research Society Conference | Budapest | 30/05 | 01/06 | 0 |
| 8th Euro-African Conference on Finance and Economics (CEAFE 2020) / Mediterranean Workshop in Economic Theory (MWET 2020) | Porto | 01/06 | 02/06 | 0 |
| 13th annual conference of the Paul Woolley Centre for the Study of Capital Market Dysfunctionality | Basel | 04/06 | 05/06 | 0 |
| 10th International Conference of the Financial Engineering and Banking Society | Chania | 04/06 | 07/06 | 0 |
| Money, Power and Print: "An Interdisciplinary Colloquium on the Financial Revolution" 2020 | Dublin | 04/06 | 06/06 | 0 |
| 11th European Banking Center Network Conference "The Evolution of the Banking Sector and Financial Stability" | Tilburg | 04/06 | 05/06 | 0 |
| 24th European Conference 2020 - Financial Management Association International | Limassol | 10/06 | 12/06 | 0 |
| Experimental Finance 2020 | Innsbruck | 10/06 | 12/06 | 0 |
| 2020 EABH Annual Meeting "Finance & Migration" - European Association for Banking and Financial History | Athens | 11/06 | 11/06 | 0 |
| Thirteenth Annual SoFiE Conference - Society for Financial Econometrics | San Diego | 15/06 | 17/06 | 0 |
| 6th Symposium on Quantitative Finance and Risk Analysis (QFRA 2020) | Crete | 24/06 | 26/06 | 0 |
| 29th Annual Meeting of the European Financial Management Association - EFMA 2020 | Dublin | 24/06 | 27/06 | 0 |
| 27th Annual Conference of the Multinational Finance Society 2020 | Gdansk | 28/06 | 01/07 | 1 |
| Conference: New Perspectives on Interwar Financial and Banking Crises | Paris | 29/06 | 30/06 | 0 |
| 11th Portuguese Finance Network Conference (PFN2020) | Braga | 29/06 | 01/07 | 0 |
| 16th EADI General Conference 2020: Solidarity, Peace, and Social Justice - European Association of  Development Research and Training Institutes | Hague | 29/06 | 02/07 | 0 |
| 28th Finance Forum, the Annual Meeting of the Spanish Finance Association (AEFIN). | Carcavelos | 02/07 | 03/07 | 0 |
| 2020 Joint Real Estate Conference by the Asian Real Estate Society (AsRES),  The Global Chinese Real Estate Congress (GCREC) Conference 2020 | Singapore | 12/07 | 15/07 | 0 |
| Healthcare Information & Management Systems Society | Orlando | 09/03 | 13/03 | 0 |
| 2020 Annual BHC Meeting - Business History Conference | Charlotte | 12/03 | 14/03 | 1 |
| GOR AG FIFI Workshop 2020 - German Operations Research Society | Halle (Saale) | 18/03 | 19/03 | 0 |
| Third ARTEM OCC Conference „Creativity, Innovation and Sustainability in the Age of Disruptions:  Dealing with Global Challenges through Trans- and Interdisciplinary Approaches“ | Chemnitz | 19/03 | 21/03 | 0 |
| The 7th International Conference on Business and Social Science (ICBASS) | Osaka | 24/03 | 26/03 | 0 |
| 47th Academy of International Business UK & Ireland Chapter Conference 2020  "Re-visiting SME and MNE Internationalization in an era of paradox" | Glasgow | 02/04 | 04/04 | 0 |
| 13th Biennial MSPL Modern Studies in Property Law Conference 2020 | Newcastle-upon-Tyne | 15/04 | 17/04 | 0 |
| XVII Circle Conference - Marketing and the New Millenium | Vitez | 15/04 | 17/04 | 0 |
| Eighth International Conference on Information Systems, Logistics and Supply Chain ILS 2020 | Austin | 22/04 | 24/04 | 0 |
| 18th European Academic Conference on Internal Audit and Corporate Governance | London | 22/04 | 24/04 | 0 |
| International Conference on Contemporary and Emerging Trends in Commerce ICCETCMC2020 | Bangalore | 23/04 | 24/04 | 0 |
| Innovation Pitch Competition IEPC2020 | San Francisco | 23/04 | 24/04 | 1 |
| 35th UN/CEFACT Forum 2020 - United Nations Centre for Trade Facilitation and e-business | Geneva | 27/04 | 01/05 | 0 |
| 1st Erasmus+ International Conference 1stEIC | Barcelos | 04/05 | 04/05 | 0 |
| 2nd Tokenomics - International Conference on Blockchain Economics, Security and Protocols | Toulouse | 11/05 | 12/05 | 0 |
| 3E Conference – ECSB Entrepreneurship Education Conference 2020 - European Council for Small Business and Entrepreneurship | Trondheim | 13/05 | 15/05 | 0 |
| 175th EAAE Seminar "The wind of change of sustainability standards: sailing economic, governance, and policy perspectives" - European Association of Agricultural Economists | Gargnano | 13/05 | 15/05 | 0 |
| 2020 AMA Global SIG Conference Sicily AMA Global SIG 2020 | Taormina | 18/05 | 21/05 | 0 |
| International Conference on Operations and Supply Chain Analytics (ICOSCA 2020) ICOSCA 2020 | Shillong | 20/05 | 22/05 | 0 |
| AMS Annual Conference AMS2020 | Coral Gables | 20/05 | 20/05 | 0 |
| 36th EAA Doctoral Colloquium 2020 - European Accounting Association | Bucharest | 23/05 | 26/05 | 0 |
| 2nd IEEE International Workshop on Smart Circular Economy (to be held virtually) SmaCE '20 | Los Angeles | 25/05 | 27/05 | 1 |
| 2020 International Conference on Business, Economics and Management in the Digital World 2020ICBEM | Taipei | 26/05 | 29/05 | 0 |
| EMAC 2020 – The 49th Annual Conference of the European Marketing Academy | Budapest | 27/05 | 29/05 | 0 |
| 43rd Annual Congress of the European Accounting Association EAA 2020 | Bucharest | 27/05 | 29/05 | 0 |
| CERALE-EGADE Business School 2020 Conference CERALE 2020 | Mexico City | 27/05 | 29/05 | 0 |
| NCSB conference - 21st Nordic Conference on Small Business Research | Kolding | 27/05 | 29/05 | 0 |
| 49th EMAC Annual Conference 2020 - European Marketing Academy | Budapest | 27/05 | 29/05 | 0 |
| New England AIS 2020 CONFERENCE NEAIS 2020 | Boston | 29/05 | 29/05 | 0 |
| The 13th IEEE international conference on Logistics and Supply Chain Management | Fez | 03/06 | 05/06 | 0 |
| 2020 Industry Studies Association Annual Conference | Boston | 03/06 | 05/06 | 1 |
| 2020 CAAA Annual Conference - Canadian Academic Accounting Association | Saskatoon | 04/06 | 06/06 | 0 |
| IABS 2020: International Association for Business and Society 2020 | Lisbon | 04/06 | 07/06 | 0 |
| 3rd Summer Academy on Cultural and Creative Industries and Local Development - 2020 | Trento | 07/06 | 12/06 | 1 |
| 23rd International Conference on Business Information Systems BIS 2020 | Colorado Springs | 08/06 | 10/06 | 1 |
| 23rd Uddevalla Symposium 2020 "The Geography of Creativity, Innovation, and Entrepreneurship  – Implications for Regional Growth and Development" | Stenungsund | 11/06 | 13/06 | 0 |
| 48th Annual Administrative Sciences Association of Canada (ASAC) Conference | St. John's | 12/06 | 15/06 | 1 |
| 14th Workshop on Accounting and Economics 2020 | Rotterdam | 18/06 | 19/06 | 0 |
| 19th European Conference on Research Methodology for Business and Management Studies ECRM 2020 | Aveiro | 18/06 | 19/06 | 1 |
| IFORS2020 conference Data Envelopment Analysis and Performance Measurement stream at | Seoul | 21/06 | 26/06 | 0 |
| GENMAC2020: GENMAC (Gender, Markets, Consumers) Conference | Stockholm | 23/06 | 24/06 | 0 |
| 9th Biennial Conference of the Academy of World Business, Marketing and Management Development | St. Catharines | 23/06 | 26/06 | 0 |
| CCT2020: Consumer Culture Theory 2020 | Leicester | 25/06 | 28/06 | 0 |
| Association of Business Historians Annual Conference 2020 "Bubbles and Crises; Mayhem and Misery; Corruption and Disruption" | Nottingham | 26/06 | 27/06 | 0 |
| EurOMA Conference 2020 "Managing Operations for Impact" | Coventry | 28/06 | 30/06 | 1 |
| 15th World Congress of Accounting Historians 2020 | St. Petersburg | 28/06 | 01/07 | 0 |
| 10th Annual Conference of the Academy of International Business (AIB) Latin America and the Caribbean Chapter | Miami | 30/06 | 01/07 | 1 |
| EDEN Doctoral Seminar on Advanced Studies in Entrepreneurship - European Institute for Advanced Studies in Management | Brussels | 30/06 | 03/07 | 0 |
| Critical Perspectives on Accounting Conference 2020 | Toronto | 06/07 | 09/07 | 1 |
| American Accounting Association 2020 Annual Meeting. | Atlanta | 07/08 | 12/08 | 1 |
| 15th International Symposium on Functional and Logic Programming | Akita | 23/04 | 24/04 | 0 |
| The Fourth International Conference on Computational Thinking Education 2020 | HongKong | 07/05 | 09/05 | 0 |
| GLSVLSI 2020: ACM Great Lakes Symposium on VLSI | Beijing | 27/05 | 29/05 | 0 |
| 14th Conference on Logic and the Foundations of Game and Decision Theory | Groningen | 08/07 | 10/07 | 0 |
| IEEE BSC 2020: IEEE International Workshop on Blockchain and Smart Contracts | Vilnius | 27/07 | 31/07 | 0 |
| VL/HCC 2020: 2020 IEEE Symposium on Visual Languages and Human-Centric Computing | Dunedin | 10/08 | 14/08 | 1 |
| MFCS 2020: 45th International Symposium on Mathematical Foundations of Computer Science | Prague | 24/08 | 28/08 | 1 |
| Euro-Par2020: Euro-Par: 26th International European Conference on Parallel and Distributed Computing | Warsaw | 24/08 | 28/08 | 1 |
| 24rd International Conference on Theory and Practice of Digital Libraries | Lyon | 25/08 | 27/08 | 1 |
| DSD 2020: Euromicro Conference on Digital System Design | Portorož | 26/08 | 28/08 | 1 |
| CONCUR20: CONCUR 2020: 31st International Conference on Concurrency Theory | Wien | 31/08 | 02/09 | 1 |
| CCC 2020: Continuity, Computability, Constructivity – From Logic to Algorithms, Faro (Portugal) | Faro | 31/08 | 04/09 | 1 |
| 49th GESIS Spring Seminar: Digital Behavioral Data - GESIS Leibniz Institute for the Social Sciences | Cologne | 02/03 | 20/03 | 0 |
| 3rd Conference of the EURO Practice of OR Working Group 2020 | Berlin | 19/03 | 20/03 | 0 |
| Research conference on "The impact of machine learning and AI on the UK economy" | London | 25/03 | 26/03 | 1 |
| 91st Meeting of EURO Working Group on Multicriteria Decision Aiding (EWG-MCDA91) 2020 | Ispra | 02/04 | 04/04 | 0 |
| Artificial Intelligence and Robotics in Service Interactions: Trends, Benefits and Challenges | Zaragoza | 02/04 | 03/04 | 0 |
| 11th bi-annual Postal Economics Conference on E-commerce, Digital Economy and Delivery Services | Toulouse | 02/04 | 03/04 | 0 |
| Web Archiving Conference | Montreal | 12/04 | 13/04 | 0 |
| 9th International Conference on "Mathematical and Statistical Methods for Actuarial Sciences and Finance" - MAF 2020 | Geneva | 15/04 | 17/04 | 0 |
| CIREQ Montreal Econometrics Conference 2020 - Centre Interuniversitaire de Recherche en Economie Quantitative | Montreal | 01/05 | 02/05 | 0 |
| 4th Workshop on Financial Econometrics and Empirical Modeling of Financial Markets | Chemnitz | 04/05 | 05/05 | 0 |
| Workshop recherche EduBIM 2020 EduBIM 2020 | Biarritz | 06/05 | 07/05 | 0 |
| The 2nd Games, Agents and Incentives Workshop @ AAMAS 2020 | Auckland | 09/05 | 13/05 | 1 |
| PV2020: Ensuring Long-Term Preservation and Adding Value to Scientific and Technical Data | Geneva | 12/05 | 14/05 | 0 |
| Financial Econometrics Conference - Toulouse School of Economics | Toulouse | 15/05 | 16/05 | 0 |
| 8th Joint Symposium on Computational Intelligence (JSCI8) | Bangkok | 21/05 | 21/05 | 1 |
| The 14th Learning and Intelligent Optimization (LION 14) Conference | Athens | 24/05 | 28/05 | 0 |
| The 2nd International Conference on Big Data in Business | Tampa | 27/05 | 30/05 | 0 |
| 19th International Workshop on Spatial Econometrics and Statistics 2020 | Nantes | 28/05 | 29/05 | 0 |
| 14th RCEA Bayesian Econometrics Workshop - Rimini Centre for Economic Analysis | Waterloo | 29/05 | 31/05 | 0 |
| 7th RCEA Time Series Econometrics Workshop - Rimini Centre for Economic Analysis | Waterloo | 29/05 | 30/05 | 0 |
| Workshop on Dimensionality Reduction and Inference in High-Dimensional Time Series UM HD-TS 2020 | Maastricht | 02/06 | 03/06 | 0 |
| 2020 Symposium on Data Science & Statistics | Pittsburgh | 03/06 | 06/06 | 1 |
| SocialSens2020: 5th International Workshop on Social Sensing: Special Edition on Narrative Analysis on Social Media | Atlanta | 08/06 | 11/06 | 1 |
| International Workshop on Cyber Social Threats | Atlanta | 08/06 | 08/06 | 1 |
| HAIW2020: Human-AI Workshop @ECAI2020 | Santiago de Compostela | 08/06 | 08/06 | 0 |
| MARBLE 2020: The 2nd International Conference on Mathematical Research for Blockchain Economy | Faro | 08/06 | 10/06 | 1 |
| 11th ECB Conference on Forecasting Techniques "Avenues for the next 20 years" - European Central Bank | Frankfurt am Main | 08/06 | 09/06 | 0 |
| 2nd International Workshop on Forecasting for Social Good | Bordeaux | 10/06 | 11/06 | 0 |
| ICR2020 meets CyberSpike | Tallinn | 12/06 | 12/06 | 0 |
| International Conference on Applied Statistics (ICAS), 14th Edition, 12-13 June 2020, Bucharest, Romania | Bucharest | 12/06 | 13/06 | 0 |
| Nipe summer school The econometrics of big data 9th edition | Braga | 15/06 | 18/06 | 0 |
| 21st Annual International Conference on Digital Government Research dg.o 2020 | Seoul | 15/06 | 19/06 | 1 |
| 2020 Econometric Society Summer School in Dynamic Structural Econometrics | Zürich | 15/06 | 21/06 | 0 |
| 2nd International Congress on Blockchain and Applications | L'Aquila | 17/06 | 19/06 | 0 |
| IPIC 2020: 7th International Physical Internet Conference | Shenzhen | 17/06 | 19/06 | 0 |
| 5th International Conference "Digital Transformation & Global Society" (DTGS 2020) - online conference | St. Petersburg | 17/06 | 19/06 | 1 |
| CI2020: Association for Computing Machinery: Collective Intelligence 2020 US | Boston | 18/06 | 19/06 | 1 |
| CI2020: Association for Computing Machinery: Collective Intelligence 2020 DK | Copenhagen | 18/06 | 19/06 | 1 |
| IoT-SenCity 2020: Internet of Things Crowdsensing for Smart Cities | Como | 22/06 | 25/06 | 1 |
| International Workshop on Trust, Ethics and Information Quality in Smart Environments (IW-TEIQ 2020) | Antwerp | 22/06 | 24/06 | 1 |
| Global Information Technology Management Association 2020 Conference (GITMA 2020) | Paris | 22/06 | 24/06 | 1 |
| 6th International AR VR Conference, Lisbon | Lisbon | 24/06 | 26/06 | 0 |
| XIV World Conference SEA 2020 - Spatial Econometrics Association | Jönköping | 24/06 | 26/06 | 0 |
| 2020 Ammersee Workshop "Natural Experiments and Controlled Field Studies“ | Ohlstadt | 26/06 | 28/06 | 0 |
| International conference on ICT enhanced SSH 2020 (ICTeSSH 2020) | Amsterdam | 29/06 | 01/07 | 1 |
| 7th Annual Conference of the International Association for Applied Econometrics IAAE 2020 | London | 30/06 | 03/07 | 0 |
| DSMP'2020: 2020 IEEE Third International Conference Data Stream Mining & Processing | Lviv | 21/08 | 25/08 | 1 |
| 26th ACM SIGKDD Conference on Knowledge Discovery and Data Mining | San Diego | 23/08 | 27/08 | 1 |
| 14th Annual Empirical Investigations in Trade and Investment Conference - Forum for Research in Empirical International Trade | Fukuoka | 05/03 | 07/03 | 0 |
| 57th Annual Meetings of the Public Choice Society 2020 | Newport Beach | 12/03 | 14/03 | 1 |
| SIEPR Economic Summit 2020 - Stanford Institute of Economic Policy Research | Stanford | 13/03 | 13/03 | 0 |
| CESifo Area Conference on Energy and Climate Economics 2020 | Munich | 13/03 | 14/03 | 0 |
| 12th Annual SBCA Conference and Meeting - Society for Benefit-Cost Analysis | Washington | 16/03 | 17/03 | 0 |
| World Bank annual Land and Poverty Conference 2020 "Institutions for Equity and Resilience" | Washington | 16/03 | 20/03 | 0 |
| XXIII 2020 Workshop in International Economics and Finance | Quito | 16/03 | 17/03 | 0 |
| Second Economic Research Southern Africa workshop on ‘Structural Constraints on the Economy, Growth and Political Economy’ | Johannesburg | 18/03 | 18/03 | 0 |
| 2020 Post-Conference Professional Development Workshops "Promoting Objectivity & Relevance in Benefit-Cost Analysis" - Society for Benefit-Cost Analysis | Washington | 18/03 | 18/03 | 0 |
| CESifo Area Conference on Public Economics 2020 | Munich | 19/03 | 21/03 | 0 |
| Conference on Adverse Health Conditions and Labor Market Policies | Amsterdam | 19/03 | 20/03 | 0 |
| 16th International WEAI Conference 2020 - Western Economic Association International | Shanghai | 19/03 | 22/03 | 0 |
| Research conference on "Financial frictions: Macroeconomic implications and policy options for emerging economies" | Santiago de Chile | 19/03 | 20/03 | 0 |
| 13th History of Recent Economics Conference - HISRECO 2020 | Lausanne | 20/03 | 21/03 | 0 |
| 9th PhD Student Conference in International Macroeconomics and Financial Econometrics 2020 | Nanterre | 20/03 | 20/03 | 0 |
| 15th Australasian Trade Workshop ATW2020 | Canberra | 21/03 | 22/03 | 0 |
| CSAE Conference 2020: Economic Development in Africa - Centre for the Study of African Economies | Oxford | 22/03 | 24/03 | 0 |
| EcoMod School of Modeling and Data Science - Dubai UAE 2020 | Dubai | 22/03 | 26/03 | 0 |
| 5th IZA Junior/Senior Symposium - Institute for the Study of Labor | Bonn | 23/03 | 24/03 | 0 |
| International Conference on "The German Labor Market in a Globalized World: Trade, Technology, and Demographics" | Mannheim | 23/03 | 24/03 | 0 |
| 89th International Atlantic Economic Conference - Rome 2020 | Rome | 25/03 | 28/03 | 0 |
| ERSA Workshop "Representivity, Diversity, and Gender in Economics in South Africa" | Ballito | 26/03 | 26/03 | 1 |
| 11th conference of the International Research Forum on Monetary Policy "Ensuring resilience – role and limitations of monetary policy" | Frankfurt am Main | 26/03 | 27/03 | 0 |
| 24th Conference "Theories and Methods in Macroeconomics" (T2M) | Marseille | 26/03 | 27/03 | 0 |
| 14th ifo Dresden Workshop on Macroeconomics & Business Cycle Research | Dresden | 27/03 | 28/03 | 0 |
| 84th Annual Meeting of the Midwest Economics Association 2020 | Evanston | 27/03 | 29/03 | 0 |
| 1st Workshop "Emerging Market MacroEconomics" - EMME 2020 | Bordeaux | 27/03 | 27/03 | 0 |
| 2020 CES North America Annual Conference - Chinese Economists Society | Cambridge | 27/03 | 28/03 | 0 |
| Association for Evolutionary Economics at the 84th Midwest Economics Association Annual Meeting 2020 | Evanston | 27/03 | 29/03 | 0 |
| 41st Annual Meeting of the Association for Institutional Thought AFIT 2020 (in conjunction with the 62nd Annual Western Social Science Association Conference) | Portland | 01/04 | 04/04 | 1 |
| 59th Annual Meetings of SRSA 2020 - Southern Regional Science Association | Savannah | 02/04 | 04/04 | 0 |
| 3rd International Conference on Gender Research ICGR 2020 | Reading | 02/04 | 03/04 | 1 |
| 6th LEER Conference on Education Economics - Leuven Economics of Education Research | Leuven | 02/04 | 03/04 | 0 |
| 2020 Royal Economic Society Annual Conference | Belfast | 06/04 | 08/04 | 0 |
| XXI April International Academic Conference on Economic and Social Development | Moscow | 06/04 | 10/04 | 1 |
| 3rd African ITS Conference 2020 - International Telecommunications Society | Cape Town | 06/04 | 07/04 | 0 |
| First Economic Research Southern Africa workshop on the ‘Economics of Digitization’ | Cape Town | 08/04 | 09/04 | 0 |
| 6th RES Symposium of Junior Researchers 2020 - Royal Economic Society | Belfast | 09/04 | 09/04 | 0 |
| 44th Annual Conference on the Political Economy of the World-System (PEWS) 2020 “World-Systems Analysis in a Critical Juncture” | Baltimore | 10/04 | 11/04 | 0 |
| 31st EBES Conference - Warsaw 2020 - Eurasia Business and Economics Society | Warsaw | 15/04 | 17/04 | 1 |
| 2020 Meeting of the of the European Public Choice Society EPCS | Lucerne | 15/04 | 18/04 | 0 |
| 29th Annual Hyman P. Minsky Conference 2020 | Blithewood | 16/04 | 16/04 | 0 |
| 17th Midwest International Economic Development Conference 2020 | West Lafayette | 17/04 | 18/04 | 1 |
| 12th Workshop on Labour Economics 2020 - Institute for Labour Law and Industrial Relations in the European Union | Trier | 17/04 | 18/04 | 0 |
| 2020 Annual Conference of the Economic History Society | Oxford | 17/04 | 19/04 | 0 |
| EABC Dating Committee Conference on Finding the Gap "Output Gap Measurement in the Euro Area" | Florence | 17/04 | 17/04 | 0 |
| JADE-CEPR-TIME-CREPE Conference on Economic Development | Tokyo | 18/04 | 19/04 | 0 |
| Euro Area Business Cycle Network Training School "Fiscal Policy: Estimating Causal Evidence and Positive Theory" | Mannheim | 20/04 | 21/04 | 1 |
| 30th Royal Economic Society Easter Training School 2020 | Colchester | 20/04 | 22/04 | 0 |
| First annual workshop on International Capital Flows and Financial Policies | Paris | 22/04 | 22/04 | 0 |
| The 14th Workshop on the Organisation, Economics and Policy of Scientific Research WOEPSR 2020 | Munich | 23/04 | 24/04 | 0 |
| 4th IZA Workshop on Gender and Family Economics | Bonn | 24/04 | 25/04 | 0 |
| 10th European Meeting of the Urban Economics Association 2020 | London | 24/04 | 25/04 | 0 |
| Scottish Economic Society Annual Conference - SES 2020 | Perth | 26/04 | 28/04 | 0 |
| Spillovers in a “low-for-long” world - Joint BIS, BoE, ECB and IMF conference | Frankfurt am Main | 27/04 | 28/04 | 0 |
| 2020 SEA Annual Meeting "Convenience" - Society for Economic Anthropology | South Bend | 30/04 | 02/05 | 1 |
| 18th Annual International Industrial Organization Conference 2020 | Philadelphia | 01/05 | 03/05 | 0 |
| Midwest Economic Theory Conference | Greenville | 01/05 | 03/05 | 0 |
| EcoMod School of Modeling and Data Science - Washington, DC 2020 | Washington | 04/05 | 08/05 | 0 |
| CEPR/EBRD/ECB Symposium on “Climate change, finance and green growth” | Frankfurt am Main | 06/05 | 06/05 | 0 |
| Euro Area Business Cycle Network (EABCN) Conference "Empirical Advances in Monetary Policy" | Paris | 06/05 | 07/05 | 0 |
| 47th Economics Conference of the OeNB "The EU’s “Northern” Enlargement 25 Years on: A Comparative Stocktaking and Outlook" | Wien | 07/05 | 08/05 | 0 |
| 10th ifo Dresden Workshop on Labor Economics and Social Policy | Dresden | 07/05 | 07/05 | 0 |
| 2020 ZEW Public Finance Conference - Centre for European Economic Research | Mannheim | 07/05 | 08/05 | 1 |
| 3rd RCEA Warsaw Money-Macro-Finance Conference (WMMFC 2020) - Rimini Centre for Economic Analysis | Warsaw | 08/05 | 09/05 | 0 |
| Frankfurt-Mannheim-Macro-Workshop 2020 | Frankfurt am Main | 08/05 | 08/05 | 0 |
| NABE-Bundesbank International Symposium 2020 | Eltville am Rhein | 11/05 | 12/05 | 0 |
| Third Joint EU Cohesion Policy Conference - Maximising the Opportunities for Cohesion Policy in Challenging Times: An Academic and Policy Debate | Zagreb | 11/05 | 13/05 | 0 |
| 2nd annual Workshop for Women in Macroeconomics, Finance and Economic History | Berlin | 12/05 | 13/05 | 0 |
| 2020 Latin American Workshop in Economic Theory | Santiago de Chile | 12/05 | 13/05 | 0 |
| 28th CEPR European Summer Symposium in International Macroeconomics (ESSIM) | Paris | 13/05 | 15/05 | 0 |
| 7th International Meeting in Law & Economics 2020 | Paris | 14/05 | 15/05 | 0 |
| Rethinking Clusters – III International Conference on Cluster Research | Valencia | 14/05 | 15/05 | 0 |
| 11th Annual Conference of the LACEA Trade, Integration and Growth Network | Buenos Aires | 14/05 | 15/05 | 0 |
| 2020 Utah Experimental Economics Conference | Salt Lake City | 14/05 | 16/05 | 1 |
| Fifth Conference Global Value Chains, Trade and Development "Trade Agreements and Supply Chains" | Brussels | 14/05 | 16/05 | 0 |
| EU and its neighbourhood: enhancing EU actorness in the eastern borderlands EURINT 2020 Conference | Iasi | 14/05 | 16/05 | 0 |
| IAB-ECSR interdisciplinary conference "Refugee Migration and Integration Revisited: Lessons from the Recent Past" | Nuremberg | 14/05 | 15/05 | 0 |
| EES Conference "New Developments in the Macroeconomics of Labor Markets" 2020 | Philadelphia | 14/05 | 15/05 | 0 |
| 1st Bordeaux Workshop on Chinese Economics 2020 | Bordeaux | 15/05 | 15/05 | 0 |
| 23rd IZA Summer School in Labor Economics 2020 - Institute for the Study of Labor | Buch/Ammersee | 15/05 | 28/05 | 0 |
| 27th International Economic Conference of Sibiu IECS 2020 | Sibiu | 15/05 | 16/05 | 0 |
| Midwest Macroeconomic Meetings (Spring 2020) | Logan | 15/05 | 16/05 | 0 |
| Health Economics Initiative Annual Conference - Becker Friedman Institute for Economics | Chicago | 15/05 | 16/05 | 0 |
| ifo & WZB Conference "Econometric Evaluation of School Reforms" | Munich | 15/05 | 16/05 | 0 |
| 8th ABFER Annual Conference 2020 - Asian Bureau of Finance and Economic Research | Singapore | 18/05 | 21/05 | 0 |
| Workshop on Labour - 2020 RIDGE May Forum | Lima | 18/05 | 19/05 | 0 |
| Workshop on Public Economics - 2020 RIDGE May Forum | Lima | 18/05 | 19/05 | 0 |
| 4th Doctoral Workshop on the Economics of Digitization | Toulouse | 19/05 | 20/05 | 0 |
| 13th Annual Meeting Impact Evaluation Network (IEN) - 2020 RIDGE May Forum | Lima | 19/05 | 20/05 | 0 |
| World Media Economics and Management Conference 2020 WMEMC 2020 | Rome | 20/05 | 24/05 | 0 |
| ESCoE Conference on Economic Measurement 2020 | London | 20/05 | 22/05 | 0 |
| Workshop on Inequality and Poverty (NIP) - 2020 RIDGE May Forum | Lima | 20/05 | 21/05 | 0 |
| Workshop the Political Economy of Conflict and Crime - 2020 RIDGE May Forum | Lima | 20/05 | 20/05 | 0 |
| 2nd LACEA BRAIN Conference - 2020 RIDGE May Forum | Montevideo | 20/05 | 22/05 | 0 |
| Sixteenth CIREQ Ph.D. Students’ Conference 2020 - Centre Interuniversitaire de Recherche en Economie Quantitative | Montreal | 20/05 | 20/05 | 0 |
| Conference "Inflation: Drivers and Dynamics 2020" | Cleveland | 21/05 | 22/05 | 1 |
| 25th Coalition Theory Network Workshop | Barcelona | 21/05 | 22/05 | 0 |
| 30th Annual Meeting of the American Law and Economics Association 2020 | Chicago | 21/05 | 22/05 | 0 |
| RIDGE/LACEA-PEG Workshop on Political Economy - 2020 RIDGE May Forum | Lima | 21/05 | 22/05 | 0 |
| 54th Annual Conference of the Canadian Economics Association 2020 | Toronto | 22/05 | 24/05 | 0 |
| 17th IZA Annual Migration Meeting - Institute for the Study of Labor IZA | Bonn | 22/05 | 23/05 | 0 |
| 2nd Annual Conference on the Economics of Crime and Justice 2020 | London | 22/05 | 23/05 | 0 |
| CESifo Area Conference on Global Economy 2020 | Munich | 22/05 | 23/05 | 0 |
| Tenth Annual AEA Conference on Teaching and Research in Economic Education (CTREE) 2020 | Chicago | 27/05 | 29/05 | 0 |
| 6th International Conference on Decision Support System Technology ICDSST 2020 | Zaragoza | 27/05 | 27/05 | 1 |
| Third Behavioral Macroeconomics Workshop: | Bamberg | 28/05 | 30/05 | 0 |
| 45th Annual Economic and Business History Society Conference 2020 | Atlanta | 28/05 | 30/05 | 0 |
| 8th Ghent University Workshop on Empirical Macroeconomics 2020 | Ghent | 28/05 | 29/05 | 0 |
| 9th Mannheim Energy Conference | Mannheim | 28/05 | 29/05 | 0 |
| 5th International Conference on Sport Economics & Sport Management (SESM) | Paris | 28/05 | 29/05 | 1 |
| Essen Health Conference 2020 "Where Health meets Labour and Education Economics" | Essen | 28/05 | 30/05 | 0 |
| 24th Annual Conference of the European Society for the History of Economic Thought (ESHET 2020) | Sofia | 28/05 | 30/05 | 0 |
| 2020 Cliometric Society Conference | Orange | 29/05 | 30/05 | 0 |
| 12th International Conference "Economies of the Balkan and Eastern European Countries" | Rijeka | 29/05 | 31/05 | 1 |
| RCEA 8th Biennial Conference "Future of Growth" - Rimini Centre for Economic Analysis | Waterloo | 29/05 | 31/05 | 0 |
| Arne Ryde Workshop "Heterogeneous Agent Models in Macroeconomics: Advances in Continuous Time" | Lund | 29/05 | 30/05 | 0 |
| CSIO-TSE 18th Joint Workshop on Industrial Organization 2020 | Toulouse | 29/05 | 30/05 | 0 |
| CESifo Area Conference on Labor Economics 2020 | Munich | 29/05 | 30/05 | 0 |
| 4th Contemporary Issues in Economy & Technology Conference CIET 2020 - University of Split | Split | 29/05 | 30/05 | 1 |
| The 16th International Symposium on Econometric Theory and Applications: SETA2020 | Seoul | 30/05 | 31/05 | 0 |
| 1st International Reading PhD Workshop in Economics - University of Reading | Reading | 01/06 | 02/06 | 0 |
| 13th World Congress of the RSAI 2020 "Smart Regions – Opportunities for sustainable development in the digital era" - Regional Science Association International | Marrakech | 02/06 | 05/06 | 0 |
| Global Sales Science Institute (GSSI) Conference 2020 "Disrupting the sales force:  Technology, Globalization and the Changing nature of work" 2020 | Montpellier | 03/06 | 06/06 | 0 |
| Euro Area Business Cycle Network Training School "Recent Developments in Forecasting" | Rome | 03/06 | 05/06 | 0 |
| 2020 AERE Annual Summer Conference - Association of Environmental and Resource Economists | Miami | 03/06 | 05/06 | 0 |
| The Micro and Macro of The Changing Nature of Work and Leisure | Warwick | 04/06 | 05/06 | 0 |
| 22nd INFER Annual Conference 2020 - INFER International Network for Economic Research | Paris | 03/06 | 05/06 | 0 |
| 16th Australasian Development Economics Workshop (ADEW 2020) | Melbourne | 04/06 | 05/06 | 0 |
| 8th IZA Workshop on Environment, Health and Labor Markets - Institute for the Study of Labor | Bonn | 04/06 | 05/06 | 0 |
| Nineteenth Annual EEFS Conference 2020 - European Economics and Finance Society | Kraków | 04/06 | 07/06 | 0 |
| 2nd Vienna Workshop on Economic Forecasting 2020 | Wien | 04/06 | 05/06 | 0 |
| XXIth AISSEC Scientific Conference "Comparative Perspectives on Economic Development and Inequalities"  - Italian Association for the Study of Comparative Economic Systems | Urbino | 04/06 | 06/06 | 0 |
| 25th Spring Meeting of Young Economists SMYE 2020 - European Association of Young Economists | Bologna | 04/06 | 06/06 | 0 |
| 16th CEUS Workshop on European Economics - Center for European Studies | Vallendar | 04/06 | 05/06 | 0 |
| Workshop Recent Advances in the Economics of Philanthropy | Berlin | 04/06 | 05/06 | 0 |
| 8th Oligo Workshop 2020 | Maastricht | 04/06 | 06/06 | 1 |
| The 9th International Conference Industrial Organization and Spatial Economics | St. Petersburg | 04/06 | 05/06 | 0 |
| Workshop "The Micro and Macro of the Changing Nature of Work and Leisure" | Coventry | 04/06 | 05/06 | 0 |
| XXIII Applied Economics Meeting | Palma De Mallorca | 04/06 | 05/06 | 0 |
| Workshop "Taxation and Informality" - Leibniz ScienceCampus MannheimTaxation | Mannheim | 05/06 | 06/06 | 0 |
| International Workshop "Immigration, Integration and Attitudes" | Mannheim | 05/06 | 06/06 | 0 |
| Economics of Aging Conference | Montreal | 05/06 | 06/06 | 0 |
| 9th ASHEcon Conference 2020 - American Society of Health Economists | St. Louis | 07/06 | 10/06 | 0 |
| European Workshop on Economic Theory - EWET 2020 | Akko | 07/06 | 09/06 | 0 |
| XVIth Danish International Economics Workshop 2020 | Aarhus | 08/06 | 09/06 | 0 |
| GEP Summer Trade Workshop “The Dynamics of Trade” - Nottingham Centre for Research on Globalisation and Economic Policy | Nottingham | 08/06 | 09/06 | 1 |
| 2020 IEB/UEA Summer School in Urban Economics | Barcelona | 08/06 | 10/06 | 0 |
| 3rd annual conference of the Baltic economic association | Tartu | 08/06 | 09/06 | 0 |
| CESifo Venice Summer Institute 2020: Emergence and Dynamics of Personality and Attitudes over the Life Cycle | Venice | 08/06 | 09/06 | 0 |
| CESifo Venice Summer Institute 2020: Media and Technology Influences on Economic Decision-Making | Venice | 08/06 | 09/06 | 0 |
| Barcelona GSE Summer Forum 2020 | Barcelona | 08/06 | 19/06 | 0 |
| Monash Environmental Economics Workshop - MEEW 2020 | Melbourne | 09/06 | 10/06 | 0 |
| Summer Institute on Bounded Rationality 2020 | Berlin | 09/06 | 17/06 | 0 |
| ECHOPPE 2020 conference The Economics of Housing and Housing Policies | Aix-en-Provence | 09/06 | 11/06 | 0 |
| 69th International Congress of the French Economic Association / 69ème Congrès de l'AFSE 2020 | Lille | 10/06 | 12/06 | 0 |
| Summer School "Economic Challenges in Enlarged Europe" ECEE 2020 | Tallinn | 10/06 | 12/06 | 1 |
| CESifo Venice Summer Institute 2020: Economics of Education and Equality of Opportunity | Venice | 10/06 | 11/06 | 0 |
| CESifo Venice Summer Institute 2020: The Role of Firms in Migration Research | Venice | 10/06 | 11/06 | 0 |
| CEPR/LSE Annual Symposium in Labour Economics 2020 | London | 11/06 | 12/06 | 1 |
| Workshop on Asymmetries, Nonlinearities, Information Effects, and Other Curios in Monetary Economics | Ankara | 11/06 | 12/06 | 0 |
| Thirteenth Annual Conference on Innovation Economics | Chicago | 11/06 | 12/06 | 0 |
| Seventeenth Annual Ljubljana Empirical Trade Conference LETC 2020 | Izola | 11/06 | 13/06 | 0 |
| Sixth annual Global Corporate Governance Colloquia GCGC Conference - Seoul 2020 | Seoul | 12/06 | 13/06 | 0 |
| CESifo Venice Summer Institute 2020: Using Novel Methods and Data in Applied Microeconomic Research | Venice | 12/06 | 13/06 | 0 |
| The Third China International Conference in Macroeconomics (CICM2020), | Beijing | 12/06 | 14/06 | 0 |
| SMS Special Conference Hangzhou 2020 " A Brave New World: Strategies for Adapting to a Changing Business Environment" - Strategic Management Society | Hangzhou | 12/06 | 14/06 | 0 |
| 2020 CES Annual China Conference - Chinese Economists Society | Wuhan | 13/06 | 14/06 | 0 |
| 20th Annual SAET Conference - Society for the Advancement of Economic Theory | Seoul | 14/06 | 17/06 | 0 |
| 12th International Conference "Economic Challenges in Enlarged Europe" ECEE 2020 | Tallinn | 14/06 | 16/06 | 0 |
| 23rd ITS Biennial Conference - International Telecommunications Society | Gothenburg | 14/06 | 17/06 | 0 |
| 33rd ERSA Summer School 2020 "Regions in transition, what implications for tourism and manufacturing?"  - European Regional Science Association | Bramois | 14/06 | 19/06 | 1 |
| 5th Rome Junior Finance Conference - Einaudi Institute for Economics and Finance | Rome | 15/06 | 16/06 | 0 |
| York Summer Workshops in Health Economic Evaluation - 2020 | York | 15/06 | 01/07 | 0 |
| 2020 CAES Annual Meeting - Canadian Agricultural Economics Society | Winnipeg | 16/06 | 19/06 | 0 |
| Arne Ryde Workshop "Gender, Fertility, and the Labor Market" | Lund | 17/06 | 18/06 | 0 |
| TeachECONference 2020 first annual virtual conference on economic education - Centre for Teaching and Learning Economics | London | 17/06 | 18/06 | 1 |
| 8th International Ioannina Meeting on Applied Economics and Finance (IMAEF) 2020 | Argostoli | 17/06 | 19/06 | 0 |
| 23rd Annual Conference on Global Economic Analysis "Global Economic Analysis Beyond 2020" | Tokyo | 17/06 | 19/06 | 1 |
| 2nd International Conference on Decision Economics DECON'20 | L'Aquila | 16/06 | 19/06 | 0 |
| 2020 RSA Annual Conference "Transformations: Relational Spaces, beyond Urban and Rural" - Regional Studies Association | Ljubljana | 17/06 | 20/06 | 0 |
| 4th Conference on Structural Dynamic Models " Methodology and applications in environmental economics and climate change" | Zürich | 18/06 | 19/06 | 1 |
| 2020 Annual Congress of the Swiss Society for Economics and Statistics | Zürich | 18/06 | 19/06 | 0 |
| 24th Annual Conference of the Society for Institutional & Organizational Economics SIOE 2020 | Cambridge | 18/06 | 20/06 | 1 |
| 34th Annual Conference of the European Society for Population Economics (ESPE), | Barcelona | 18/06 | 20/06 | 0 |
| 47th Annual Meeting of the History of Economics Society 2020 | Utrecht | 18/06 | 21/06 | 1 |
| Data Envelopment Analysis International Conference DEAIC 2020 | Seoul | 19/06 | 21/06 | 0 |
| 11th International Workshop on Applied Economics of Education - IWAEE 2020 | Catanzaro | 21/06 | 23/06 | 0 |
| Society for Economic Dynamics (SED) 2020 | Barcelona | 21/06 | 23/06 | 0 |
| 43rd IAEE European Conference - Paris 2020 - International Association for Energy Economics | Paris | 21/06 | 24/06 | 0 |
| 4th IZA Labor Statistics Workshop on the Measurement of Labor Market Conditions - Institute for the Study of Labor | Bonn | 22/06 | 23/06 | 0 |
| 5th Rome Junior Conference on Applied Microeconomics - Einaudi Institute for Economics and Finance | Rome | 22/06 | 23/06 | 0 |
| Fourth Biennial Conference "Empirical Investigations in Services Trade" EIST 2020 | Ankara | 22/06 | 23/06 | 0 |
| Conference on Sustainability and Competitiveness - Competitiveness Research Network (CompNet) | Bratislava | 22/06 | 23/06 | 1 |
| 6th Annual Macroprudential Conference | Amsterdam | 22/06 | 23/06 | 0 |
| Conference on the Distribution of Income and Wealth, and the Future of the Welfare State | Berlin | 22/06 | 23/06 | 0 |
| 20th Conference of the IAFEP 2020 (online conference) - International Association for the Economics of Participation | San Diego | 22/06 | 23/06 | 1 |
| Counterfactual Methods for Policy Impact Evaluation 2020 | Mannheim | 22/06 | 24/06 | 0 |
| 2020 Risk, Uncertainty and Decision RUD Conference | London | 22/06 | 24/06 | 0 |
| ITEA Annual Conference 2020 - International Transportation Economics Association | Toulouse | 22/06 | 26/06 | 0 |
| 3rd Workshop on Macroeconomic Research | Kraków | 23/06 | 24/06 | 0 |
| Sixth annual Workshop on Relational Contracts 2020 - New Economic School | Moscow | 23/06 | 24/06 | 1 |
| 5th International Conference Economics & Philosophy 2020 "Culture and Economics" | Warsaw | 23/06 | 25/06 | 0 |
| IAMO Forum 2020 "Digital transformation – towards sustainable food value chains in Eurasia" - Online Conference | Halle (Saale) | 24/06 | 26/06 | 1 |
| Fourteenth International Conference on Game Theory and Management 2020 | St. Petersburg | 24/06 | 26/06 | 0 |
| IIAS 2020 Conference "Public Governance for Climate Action" - International Institute of Administrative Sciences | Brussels | 24/06 | 26/06 | 0 |
| 25th Annual Conference of the European Association of Environmental and Resource Economists 2020 | Berlin | 24/06 | 27/06 | 1 |
| European Population Conference (EPC 2020) - European Association for Population Studies | Padova | 24/06 | 27/06 | 0 |
| 10th ILERA Regional Congress for the Americas 2020 - International Labor and Employment Relations Association | Toronto | 24/06 | 27/06 | 1 |
| 5th Biennial Work and Family Researchers Network Conference 2020 | New York | 24/06 | 27/06 | 1 |
| 14th Annual AAWE Conference in Verona | Verona | 24/06 | 28/06 | 0 |
| 2nd LSE Workshop on Political Economy of Turkey - European Institute and the Systemic Risk Centre | London | 25/06 | 26/06 | 0 |
| Thirteenth Vietnam Economist Annual Meeting - VEAM 2020 | Binh Duong | 25/06 | 26/06 | 0 |
| Annual International Conference of the German Economic Association - Research Group on Development Economics, 25-26 June 2020, Hamburg | Hamburg | 25/06 | 27/06 | 0 |
| 8th Annual Conference of the Leibniz Institute for East and Southeast European Studies (IOS),  Regensburg "Infrastructure in East and Southeast Europe in Comparative Perspective | Regensburg | 25/06 | 27/06 | 0 |
| EALE SOLE AASLE World Conference Berlin 2020 | Berlin | 25/06 | 27/06 | 1 |
| 3rd Evidence-Based Economics (EBE) Summer Meeting 2020 | Herrsching | 25/06 | 27/06 | 0 |
| 2020 Korean Association for Public Administration International Conference  “Inclusive Society, Sustainable Development, and Administrative Responsibility” | Gangneung | 25/06 | 27/06 | 0 |
| 29th IAFFE Annual Conference 2020 - International Association for Feminist Economics | Quito | 25/06 | 27/06 | 0 |
| 2020 WIDER Development Conference - World Institute for Development Economics Research | Helsinki | 26/06 | 27/06 | 0 |
| CEMIR Junior Economist Workshop on Migration Research - Center of Excellence for Migration and Integration Research | Munich | 26/06 | 27/06 | 0 |
| 10th Annual Meetings of the Armenian Economic Association 2020 (online conference) | Yerevan | 26/06 | 27/06 | 1 |
| 94th Annual WEAI Conference 2020 - Western Economic Association International | Denver | 26/06 | 30/06 | 1 |
| IBEFA Summer Meeting (in conjunction with the 95th Annual Western Economic Association International Conference) - International Banking, Economics, and Finance Association | Denver | 26/06 | 30/06 | 0 |
| 11th Annual Workshop on Economics of Risky Behavior | Honolulu | 27/06 | 28/06 | 0 |
| 2020 ThReD Conference | Namur | 27/06 | 28/06 | 0 |
| 7th Ce2 Workshop 2020 | Warsaw | 29/06 | 30/06 | 0 |
| 20th Biennial Conference of the International Institute of Fisheries Economics and Trade (IIFET 2020) | Vigo | 29/06 | 03/07 | 0 |
| Barcelona GSE Labor Economics Summer School 2020 | Barcelona | 30/06 | 03/07 | 0 |
| Conference "Inflation Dynamics, Expectations, and Targeting" | Jerusalem | 30/06 | 01/07 | 0 |
| 21st International Conference on Cultural Economics ACEI2020 | Lille | 30/06 | 03/07 | 0 |
| Barcelona GSE Labor Economics Summer School 2020 | Barcelona | 29/06 | 03/07 | 0 |
| EDEN Doctoral Seminar on Advanced Studies in Entrepreneurship - European Institute for Advanced Studies in Management | Brussels | 29/06 | 03/07 | 0 |
| 20th Biennial Conference of the International Institute of Fisheries Economics and Trade (IIFET 2020) | Vigo | 29/06 | 03/07 | 0 |
| Barcelona GSE Banking Summer School 2020 | Barcelona | 29/06 | 10/07 | 0 |
| Barcelona GSE Microeconometrics Summer School 2020 | Barcelona | 29/06 | 10/07 | 0 |
| Barcelona GSE Macroeconometrics Summer School 2020 | Barcelona | 29/06 | 17/07 | 0 |
| 21st International Conference on Cultural Economics ACEI2020 | Lille | 30/06 | 03/07 | 0 |
| 15th annual conference - Warsaw International Economic Meeting - WIEM 2020 Conference (online event) | Warsaw | 01/07 | 02/07 | 1 |
| Online Symposium on Circular Economy and Sustainability - International Network for Economic Research | Alexandroupolis | 01/07 | 03/07 | 0 |
| XXXII Villa Mondragone International Economic Seminar 2020, "The Digital Transformation and the future of Capitalism. Innovation, Well-being and Sustainable Growth" | Rome | 01/07 | 03/07 | 0 |
| XXV Workshop on Dynamic Macroeconomics | Pontevedra | 01/07 | 03/07 | 0 |
| 16th European Meeting on Game Theory (SING 16) | Reggio Calabria | 01/07 | 03/07 | 0 |
| The Foundations of Utility and Risk Conference (FUR) | Sydney | 01/07 | 04/07 | 0 |
| 18th ZEW Conference on the Economics of Information and Communication Technologies - | Mannheim | 02/07 | 02/07 | 1 |
| 11th Summer Conference of the German-speaking Section (GfR) of the European Regional Science Association (ERSA), „Structural change in rural and urban economies“ | Braunschweig | 02/07 | 03/07 | 0 |
| Princeton/Warwick/Utah Political Economy Conference 2020 | Rome | 02/07 | 03/07 | 0 |
| CEPR/Warwick/Princeton/Utah Political Economy Conference 2020 | Rome | 02/07 | 03/07 | 0 |
| 36th EGOS Colloquium 2020 - European Group for Organizational Studies - virtual event | Hamburg | 02/07 | 04/07 | 1 |
| International Conference on Applied Economics ICOAE 2020 - Virtual Conference | Crete | 02/07 | 04/07 | 1 |
| 2020 Annual Conference of the Academy of International Business AIB - Online Event | Miami | 02/07 | 06/07 | 1 |
| 14th Annual Meeting of the Portuguese Economic Journal 2020 | Porto | 03/07 | 05/07 | 0 |
| 22nd Annual Conference of the Association for Heterodox Economics "Heterodox Economics Globally" - online event | Milton Keynes | 03/07 | 05/07 | 1 |
| RIDGE Summer School in Economics (in the framework of the XIX International Economic Association IEA Congress) | Bali | 03/07 | 07/07 | 0 |
| 4th Eastern Conference on Football Economics 6th Western Conference on Football and Finance | St. Petersburg | 03/07 | 03/07 | 1 |
| Belgrade Young Economists Conference (BYEC 2020) | Belgrade | 03/07 | 04/07 | 0 |
| 40th International Symposium on Forecasting (ISF 2020) | Rio de Janeiro | 05/07 | 08/07 | 1 |
| 6th Early-Career Behavioral Economics Conference - ECBE 2020 | Bonn | 06/07 | 07/07 | 1 |
| 12th Joint IOS/APB/EACES Summer Academy on Central and Eastern Europe 2020  "Inequalities and Redistribution in Central and Eastern Europe" | Regensburg | 06/07 | 08/07 | 1 |
| PhD summer schools on Multiple Criteria Decision Aiding/Making (MCDA/MCDM) | Ankara | 06/07 | 17/07 | 0 |
| 3rd Bank of Russia International Research Conference "Monetary Policy, (Macro-) Prudential Regulation and Their Interactions" 2020 | St. Petersburg | 07/07 | 08/07 | 0 |
| European Health Economics Association Conference - EuHEA 2020 | Oslo | 07/07 | 10/07 | 0 |
| International Conference on Economic Modeling and Data Science (EcoMod2020) | Milan | 08/07 | 10/07 | 1 |
| 2020 Summer Workshop: The post-crisis housing market and financial stability: Recovery without Affordability? | HongKong | 09/07 | 10/07 | 1 |
| 14th International German Socio-Economic Panel User Conference (SOEP2020) "Inequalities and Their Subjective Perceptions in a Changing World" | Berlin | 09/07 | 10/07 | 0 |
| Australian Conference of Economists (ACE2020) "Wealth & Wellbeing " | Perth | 08/07 | 10/07 | 0 |
| 19th IZA/SOLE Transatlantic Meeting for Labor Economists (TAM) - Online Event | Buch/Ammersee | 09/07 | 10/07 | 1 |
| Australian Conference of Economists 2020 | Perth | 08/07 | 10/07 | 0 |
| 21st International Conference on Public Economic Theory (PET 2020) - Association for Public Economic Theory | Montreal | 08/07 | 11/07 | 0 |
| 10th EBHA Doctoral Summer School 2020 "Challenges for Business History in a Changing World" - European Business History Association | Barcelona | 08/07 | 11/07 | 0 |
| ISAFE 2020 Conference - International Society for the Advancement of Financial Economics | Berlin | 09/07 | 11/07 | 0 |
| 21st International Conference on Public Economic Theory (PET 2020) - Association for Public Economic Theory | Montreal | 08/07 | 11/07 | 0 |
| 10th EBHA Doctoral Summer School 2020 "Challenges for Business History in a Changing World" - European Business History Association | Barcelona | 08/07 | 11/07 | 0 |
| ISAFE 2020 Conference - International Society for the Advancement of Financial Economics | Ho Chi Minh city | 09/07 | 11/07 | 0 |
| 10th EIASM Conference on Current Research in Taxation - European Institute for Advanced Studies in Management (online event) | Brussels | 13/07 | 14/07 | 1 |
| Sixteenth Annual Conference of the Asia-Pacific Economic Association APEA 2020 | Beijing | 13/07 | 14/07 | 0 |
| Barcelona GSE Development Economics Summer School 2020 | Barcelona | 13/07 | 17/07 | 0 |
| Barcelona GSE Digital Economy Summer School 2020 | Barcelona | 13/07 | 17/07 | 0 |
| Twenty-First ACM Conference on Economics and Computation (EC'20) - online event | Budapest | 13/07 | 17/07 | 1 |
| Inequalities in Health and Healthcare - Tinbergen Institute Amsterdam | Rotterdam | 13/07 | 17/07 | 0 |
| 5th Biennial Work and Family Researchers Network Conference 2020 (Virtual Conference) | New York | 17/07 | 17/07 | 1 |
| SASE Annual Conference 2020 "Development Today: Accumulation, Surveillance, Redistribution" - Society for the Advancement of Socio-Economics (Virtual Conference) | Amsterdam | 18/07 | 20/07 | 1 |
| SABE 2020 Annual International Conference - Society for the Advancement of Behavioral Economics | Moscow | 20/07 | 26/07 | 1 |
| CESifo Area Conference on Macro, Money, and International Finance 2020 (online event) | Munich | 23/07 | 24/07 | 1 |
| 8th Summer School of the Econometric Society 2020 | HongKong | 27/07 | 31/07 | 0 |
| WFI Summer School 2020 "Firms in Society" - Ingolstadt School of Management | Ingolstadt | 27/07 | 07/08 | 0 |
| Health Economists’ Study Group Summer 2020 Meeting | Oxford | 29/07 | 31/07 | 0 |
| 31st CEPR/Study Center Gerzensee European Summer Symposium in Financial Markets (ESSFM) 2020 | Gerzensee | 20/07 | 31/07 | 0 |
| 17th PRSCO Summer Institute "Emerging Technologies in Regional Development” - Pacific Regional Science Conference Organization | Ho Chi Minh city | 29/07 | 31/07 | 0 |
| 9th GESIS Summer School in Survey Methodology 2020 - GESIS Leibniz Institute for the Social Sciences (Online Seminar) | Mannheim | 30/07 | 21/08 | 1 |
| 6th Banff Empirical Microeconomics Workshop | Banff | 31/07 | 01/08 | 0 |
| 15th Advanced Summer School in Economics and Econometrics 2020 (online event) | Crete | 02/08 | 09/08 | 1 |
| Third Essen Economics of Mental Health Workshop 2020 | Essen | 03/08 | 04/08 | 1 |
| 4th World Risk and Insurance Economics Congress (WRIEC) 2020 - online conference | New York | 04/08 | 05/08 | 1 |
| 32nd EBES Conference - Istanbul 2020 - Eurasia Business and Economics Society | Istanbul | 05/08 | 07/08 | 1 |
| Economic Theory Conference VI - Becker Friedman Institute for Economics | Chicago | 06/08 | 07/08 | 0 |
| 69th MFA Annual Conference 2020 - Midwest Finance Association | Chicago | 06/08 | 08/08 | 1 |
| 80th Annual Meeting of the Academy of Managemet 2020 (virtual event) | Vancouver | 07/08 | 11/08 | 1 |
| 2020 AAEA Annual Meeting - American Agricultural Economic Association (online meeting) | Kansas | 10/08 | 11/08 | 1 |
| PEWS Mini-Conference: The Legacies of Immanuel Wallerstein | San Francisco | 11/08 | 12/08 | 0 |
| Korean Economic Review International Conference | Seoul | 13/08 | 14/08 | 1 |
| 2020 CES North America Annual Conference (virtual conference) - Chinese Economists Society | Cambridge | 13/08 | 15/08 | 1 |
| 6th IWH-FIN-FIRE Workshop on "Challenges to Financial Stability" | Halle (Saale) | 17/08 | 18/08 | 1 |
| FRIC'20 Conference on Financial Frictions - Center for Financial Frictions | Copenhagen | 17/08 | 18/08 | 0 |
| 4th SAFE Market Microstructure Conference - Sustainable Architecture for Finance in Europe | Frankfurt am Main | 17/08 | 18/08 | 1 |
| Workshop on Finance and Politics | Helsinki | 17/08 | 18/08 | 1 |
| EDEN Doctoral Seminar on Perspectives on Projects (online event) | Brussels | 17/08 | 19/08 | 1 |
| 12th World Congress of the Econometric Society 2020 (virtual World Congress) | Milan | 17/08 | 21/08 | 1 |
| 10th annual Crash Course in Experimental Economics - Tinbergen Institute Amsterdam (online event) | Amsterdam | 17/08 | 22/08 | 1 |
| Econometric Methods for Forecasting and Data Science with Applications in Finance, Economics and Business - Tinbergen Institute Amsterdam | Amsterdam | 17/08 | 22/08 | 0 |
| HEDG online summer school "Historical Perspectives on Current Economic Issues: Big Data and Applications" - Historical Economics and Development Group | Odense | 17/08 | 28/08 | 1 |
| 2020 EFA Doctoral Tutorial - European Finance Association (online event) | Helsinki | 19/08 | 19/08 | 1 |
| 76th Annual Congress of the International Institute of Public Finance - IIPF 2020 | Reykjavik | 19/08 | 21/08 | 1 |
| 47th EFA Annual Meeting 2020 - European Finance Association (online conference) | Helsinki | 19/08 | 22/08 | 1 |
| 2020 Annual International Journal of Central Banking Research Conference "Structural changes in the financial system: new theory and evidence" | Copenhagen | 20/08 | 21/08 | 1 |
| Cedefop, Eurofound and IZA Conference on Workplace and Management Practices | Brussels | 20/08 | 21/08 | 1 |
| Fourth edition of the Salento Macro Meetings | Lecce | 21/08 | 22/08 | 0 |
| 2020 Conference on Markets and Economies with Information Frictions | Shenzhen | 22/08 | 24/08 | 0 |
| Conference on “Consumer Behavior in Financial Markets” 2020 - Swedish House of Finance | Stockholm | 24/08 | 26/08 | 1 |
| 35th Annual Congress of the European Economic Association - EEA2020 (online conference) | Rotterdam | 24/08 | 27/08 | 1 |
| EDEN Doctoral Seminar on Writing as Craft and Vocation in Marketing and Consumer Research - European Institute for Advanced Studies in Management | Espoo | 24/08 | 28/08 | 0 |
| 21th International Symposium on Inventories 2020 - International Society for Inventory Research | Budapest | 24/08 | 28/08 | 0 |
| Behavioral Macro and Complexity (online event) - Tinbergen Institute Amsterdam | Amsterdam | 24/08 | 28/08 | 1 |
| Thirteenth Annual Conference on Innovation Economics | Chicago | 27/08 | 28/08 | 1 |
| TI-BDS Joint Conference: Comparative Analysis of Enterprise Data - CAED 2020 | Rotterdam | 27/08 | 29/08 | 0 |
| 13th International Conference on Knowledge Science, Engineering and Management - KSEM 2020 | Hangzhou | 28/08 | 30/08 | 1 |
| 7th Annual Conference of the Society for Economic Measurement 2020 | Banff | 28/08 | 30/08 | 0 |
| 47th Annual Conference of the European Association for Research in Industrial Economics - EARIE 2020 (online event) | Bologna | 28/08 | 30/08 | 1 |
| 3rd Endless Summer Conference on "Financial Intermediation and Corporate Finance" | Athens | 31/08 | 01/09 | 0 |
| 5th Household Finance Workshop - SAFE Research Center „Sustainable Architecture for Finance in Europe“ | Hösbach | 31/08 | 01/09 | 0 |
| EFMD Annual Conference "Embracing the Future: A New Mandate for Business Schools" (online event) - European Foundation for Management Development | Lyon | 31/08 | 02/09 | 1 |
| BAM Doctoral Symposium 2020 (online event) - British Academy of Management | London | 01/09 | 01/09 | 1 |
| 4th REECAP meeting 2020 (webinar) - Research Network on Economic Experiments for the Common Agricultural Policy | Worldwide | 01/09 | 02/09 | 1 |
| 8th IZA Workshop on Environment, Health and Labor Markets - Institute for the Study of Labor | Bonn | 02/09 | 03/09 | 0 |
| STRATUS 2020: Systems and Technologies for Remote Sensing Applications through Unmanned Aerial Systems | Buffalo | 18/05 | 20/05 | 0 |
| IAI2020: International Congress and Workshop on Industrial AI 2020 | Luleå | 25/05 | 28/05 | 0 |
| 15th IEEE International Symposium on Broadband Multimedia Systems and Broadcasting 2020, the 15th | Paris | 02/06 | 05/06 | 1 |
| ETHICOMP 2020: 18th International Conference on the Ethical and Social issues in Information and Communication Technolgies | La Rioja | 17/06 | 19/06 | 1 |
| ​12t​h International ACM Conference on Web Science in 2020 (#WebSci20) | Southampton | 06/07 | 12/07 | 1 |
| 2nd Crowdworking Symposium "Ability - Motivation - Opportunities for Digital Work" | Paderborn | 09/07 | 10/07 | 0 |
| IEEE SmartDataServices 2020: 2020 IEEE International Conference on Smart Data Services | Beijing | 20/07 | 24/07 | 1 |
| SMSociety'20: International Conference on Social Media & Society | Chicago | 20/07 | 26/07 | 1 |
| PEARC20 conference: Practice and Experience in Advanced Research Computing 2020 | Portland | 26/07 | 30/07 | 1 |
| COINS2020: International Conference on Omni-layer Intelligent systems | Barcelona | 27/07 | 29/07 | 1 |
| IAS-16: 16th International Conference on Intelligent Autonomous System | Singapore | 28/07 | 31/07 | 0 |
| The 29th International Conference on Computer Communications and Networks (ICCCN 2020) | Honolulu | 03/08 | 06/08 | 1 |
| SEIT2020: 10th International Conference on Sustainable Energy Information Technology | Leuven | 09/08 | 12/08 | 0 |
| AUTOMATA2020: 26th International Workshop on Cellular Automata and Discrete Complex Systems | Stockholm | 10/08 | 12/08 | 1 |
| DRS2020: Design Research Society Biennial Conference 2020 | Brisbane | 11/08 | 14/08 | 1 |
| AiIC2020: The 2nd Applied Informatics International Conference | Melaka | 12/08 | 13/08 | 1 |
| 18th Annual International Conference on Privacy, Security and Trust (PST2020) | Toronto | 17/08 | 19/08 | 0 |
| SISSY2020: Workshop on Self-Improving System Integration ACSOS 2020 | Washington | 17/08 | 21/08 | 1 |
| ICPP-EMS 2020: The 2020 International Workshop on Embedded Multicore Systems | Edmonton | 17/08 | 20/08 | 1 |
| ICSGSC 2020: The 4th. IEEE International Conference on Smart Grid and Smart Cities | Osaka | 18/08 | 21/08 | 1 |
| SwACon2020: Software Assurance Conference | Arlington | 24/08 | 24/08 | 1 |
| ARES 2020: The 15th International Conference on Availability, Reliability and Security (ARES 2020) | Dublin | 25/08 | 28/08 | 1 |
| The 16th International Symposium on Open Collaboration | Madrid | 25/08 | 27/08 | 1 |
| ICS-CSR'20: 7th International Symposium for ICS & SCADA Cyber Security Research 2020 | Dublin | 26/08 | 27/08 | 0 |
| QAVS 2020: First Workshop on Quantitative Aspects of Variant-rich Systems | Wien | 31/08 | 05/09 | 1 |
| 7th International Workshop on Evolving Security & Privacy Requirements Engineering | Zürich | 31/08 | 01/09 | 1 |
| EGOV-CeDEM-ePart 2020 conference | Linkoping | 31/08 | 02/09 | 1 |
| 2020 EFMD – HUMANE Winter School "Developing the Leadership Potential of Senior University Managers" | Barcelona | 15/03 | 20/03 | 0 |
| 11th EIASM Workshop on Top Management Teams and Business Strategy Research - European Institute for Advanced Studies in Management | Paris | 26/03 | 27/03 | 0 |
| 7th Annual Symposium on Management and Social Sciences | Danang | 01/04 | 03/04 | 0 |
| Global Conference on Services and Retail Management (GLOSERV 2020) | Naples | 07/04 | 10/04 | 0 |
| 8th International OFEL Conference on Governance, Management and Entrepreneurship "From Corporations to Social Entrepreneurs: Exploring the Different Faces of Social Innovation" | Dubrovnik | 17/04 | 18/04 | 1 |
| 2020 EFMD MBA Conference "Designing the MBA Journey" - European Foundation for Management Development | St. Gallen | 19/04 | 21/04 | 0 |
| International Research Society for Public Management | Tampere | 22/04 | 24/04 | 0 |
| 9th International Scientific Conference on Project Management in the Baltic States "Project Management Development – Practice and Perspectives" | Riga | 23/04 | 24/04 | 0 |
| 35th Workshop on Strategic Human Resource Management 2020 - European Institute for Advanced Studies in Management | Braga | 23/04 | 24/04 | 0 |
| International Conference on Contemporary Issues in Management ICCIM 2020 | Greater Noida | 02/05 | 02/05 | 0 |
| Visioneers And Venturers Academic Conference 2020 VVC 2020 | Utica | 15/05 | 16/05 | 0 |
| EDEN Doctoral Seminar on "Qualitative Methods in Business Research" - European Institute for Advanced Studies in Management | Brussels | 18/05 | 21/05 | 1 |
| 20th Management International Conference (MIC) 2020 | Ljubljana | 27/05 | 30/05 | 0 |
| NASSM 2020 Conference North American Society for Sport Management | San Diego | 27/05 | 30/05 | 1 |
| emma2020: The European Media Management Association annual conference: "Contextualizing Media Management Research" | Jönköping | 03/06 | 06/06 | 1 |
| EFMD Annual Conference "Embracing the Future: A New Mandate for Business Schools" - European Foundation for Management Development | Lyon | 07/06 | 09/06 | 0 |
| XXXI ISPIM Innovation Conference "Innovating Our Common Future" - International Society for Professional Innovation Management | Berlin | 07/06 | 10/06 | 1 |
| 27th Innovation and Product Development Management Conference (IPDMC 2020) | Antwerp | 08/06 | 09/06 | 1 |
| 20th EURAM Annual Conference 2020 "The Business of Now: the future starts here" - European Academy of Management | Dublin | 10/06 | 12/06 | 0 |
| HHL Energy Conference 2020 - HHL Leipzig Graduate School of Management | Leipzig | 11/06 | 12/06 | 0 |
| DRUID20 Silver Anniversary Conference | Copenhagen | 15/06 | 17/06 | 0 |
| EDEN Doctoral Seminar on Comparative Societal (Culture) Research about Organizations - European Institute for Advanced Studies in Management | Brussels | 16/06 | 18/06 | 0 |
| ESMT Annual Forum 2020 "The New Work Illusion?" - European School of Management and Technology Berlin | Berlin | 18/06 | 18/06 | 0 |
| DGD-Jahrestagung 2020 „Demography in Central and Eastern Europe – Demographic behaviour since 1990 | Dresden | 11/03 | 13/03 | 0 |
| 13th European Social Science History Conference - ESSHC 2020 | Leiden | 18/03 | 21/03 | 0 |
| ETUI Conference "New beginnings? The European Pillar of Social Rights and the new Commission" - European Trade Union Institute | Brussels | 24/03 | 24/03 | 0 |
| American Society for Environmental History | Ottawa | 25/03 | 29/03 | 0 |
| The Pacific Sociological Association The Pacific Sociological Association’s 91st Annual Conference | Eugene | 26/03 | 29/03 | 0 |
| ERF 26th Annual Conference on Sustainable Development Goals (SDGs) as a Framework for MENA’s Development Policy | Luxor | 29/03 | 31/03 | 0 |
| IACS 2020: IACS Summit 2020 | St. Petersburg FL | 01/04 | 06/04 | 0 |
| 62nd Annual Conference of the Western Social Science Association 2020 | Portland | 01/04 | 04/04 | 1 |
| American Society for Public Administration | Anaheim | 03/04 | 07/04 | 0 |
| Political Studies Association | Edinburgh | 06/04 | 08/04 | 0 |
| 1st Asia Pacific Conference on Sustainable Development of Energy, Water and Environment System SDEWES | Gold Coast | 06/04 | 09/04 | 1 |
| 10th LCSR International Workshop | Moscow | 06/04 | 10/04 | 0 |
| American Association of Geographers (AAG) Annual Meeting | Denver | 06/04 | 10/04 | 1 |
| Western Political Science Association | Los Angeles | 09/04 | 11/04 | 0 |
| ESPAnet Austria 4th Social Policy Research Conference 2020 / 4. Forschungskonferenz von ESPAnet Austria | Graz | 16/04 | 17/04 | 0 |
| Midwest Political Science Association Annual Conference 75th Annual Conference | Chicago | 16/04 | 19/04 | 0 |
| Global Solutions Summit 2020 | Berlin | 20/04 | 21/04 | 1 |
| British Sociological Association Annual Conference | Birmingham | 21/04 | 23/04 | 0 |
| 35th Annual Conference of the Society for Industrial and Organizational Psychology - SIOP 2020 | Austin | 23/04 | 25/04 | 1 |
| Fourth GlobalFood Symposium 2020 | Göttingen | 24/04 | 25/04 | 0 |
| OIS Research Conference 2020: Open Innovation in Science (OIS) Research Conference 2020 | Geneva | 07/05 | 08/05 | 0 |
| Climate Adaptation Research Symposium 2020. Measuring and Addressing Societal Impacts . | Los Angeles | 08/05 | 08/05 | 0 |
| Neuroethics: New Ways of Investigating the Brain | Milan | 13/05 | 15/05 | 0 |
| Latin American Studies Association | Guadalajara | 13/05 | 16/05 | 1 |
| EASS 2020 Conference Sport, Diversity and Social Change | Southampton | 18/05 | 21/05 | 0 |
| LIBRARIES IN THE DIGITAL AGE (LIDA) 2020 | Dubrovnik | 19/05 | 22/05 | 0 |
| Seventh International Meeting on Experimental and Behavioral Social Sciences (IMEBESS 2020) | Lyon | 28/05 | 30/05 | 0 |
| The Law and Society Association 2020 Annual Meeting | Denver | 28/05 | 31/05 | 1 |
| PD2020: PROMETHEE Days 2020 | Rabat | 02/06 | 04/06 | 1 |
| XL Sunbelt Social Networks Conference of the International Network for Social Network Analysis - INSNA 2020 | Paris | 02/06 | 07/06 | 1 |
| 4th International Conference "Green Cities - Green Logistics for Greener Cities" GreenCities2020 | Szczecin | 03/06 | 05/06 | 0 |
| Conference on Education and Decent Work for Youth (LELAM2020) | Zürich | 04/06 | 05/06 | 0 |
| 2020 University-Industry Interaction Conference | Budapest | 09/06 | 11/06 | 1 |
| UD2020: International Conference on Universal Design 2020 | Espoo | 15/06 | 17/06 | 0 |
| SLCA2020: 7th international conference on Social Life Cycle Assessment: Impacts, Interests, Interactions | Göteborg | 15/06 | 17/06 | 1 |
| Monitoring of Mediterranean coastal areas: Problems and measurement techniques Eighth Symposium | Livorno | 16/06 | 18/06 | 0 |
| Development Studies Association Conference DSA2020: New Leadership for Global Challenges | Birmingham | 16/06 | 19/06 | 1 |
| British International Studies Association | Newcastle-upon-Tyne | 17/06 | 19/06 | 0 |
| City and Complexity | London | 17/06 | 19/06 | 1 |
| UKSS 2020: 21st UK Systems Society International Conference 2020 | Lanchaster | 21/06 | 22/06 | 0 |
| 6th International EcoSummit 2020 " Building a sustainable and desirable future: Adapting to a changing land and sea-scape" | Gold Coast | 21/06 | 25/06 | 0 |
| Council for European Studies 2020 conference | Reykjavik | 22/06 | 24/06 | 0 |
| The 9th International Conference on Social Science and Business (ICSSB) | Okinawa | 23/06 | 25/06 | 0 |
| 10th International Interdisciplinary Conference, Sustainable Development in Africa | Nairobi | 24/06 | 27/06 | 0 |
| Technology, the city and history | Canterbury | 29/06 | 30/06 | 1 |
| Helix - Regional Competitiveness, Tourism Innovation and Knowledge Transfer | Funchal | 29/06 | 01/07 | 1 |
| City Space Transformation: Renovation of The Urban Environment IPHS 2020 | Moscow | 05/07 | 08/07 | 0 |
| World Symposium on Transport and Land Use Research 2020 | Portland | 13/07 | 16/07 | 0 |
| AIMinScience 2020: 1st International Workshop on Assessing Impact and Merit in Science | Lyon | 25/08 | 25/08 | 1 |
| 9th Symposium of the European Association for Research in Transportation (hEART 2020), Lyon | Lyon | 01/09 | 03/09 | 0 |

Source: see text.
